# Sonoselective Transfection of Glioma Endothelium

**DOI:** 10.1002/advs.76997

**Published:** 2026-08-05

**Authors:** Anna C. Debski, Catherine M. Gorick, Victoria R. Breza, Jackson Tirrell, Krishan Perumal, Katherine M. Nowak, Ji Song, Natasha D. Sheybani, Richard J. Price

**Affiliations:** ^1^ Department of Biomedical Engineering University of Virginia Charlottesville Virginia USA; ^2^ Department of Radiology & Medical Imaging University of Virginia Charlottesville Virginia USA

**Keywords:** brain tumor, cancer research, endothelial dysfunction, endothelium, focused ultrasound, genetic enhancement, glioma transfection, tropism, tumor microenvironment

## Abstract

The blood‐brain and blood‐tumor barriers impede therapeutic delivery to glioblastoma (GBM). Furthermore, dysfunctional endothelial cells in GBM enable an immunosuppressive tumor microenvironment (TME) and enhance therapeutic resistance. However, the significance of endothelial dysfunction for GBM progression also positions the endothelium as a rich target for gene therapy. Here, we leveraged the presence of exofacial thiols (SH) on cells within the TME and developed polymeric, plasmid‐bearing, densely PEGylated, polyethyleneimine nanoparticles (NPs) functionalized with free thiol groups to facilitate targeting and transfection (SH‐NPs). Delivering SH‐NPs to GBM with focused ultrasound (FUS) elicited exceptionally high tropism for GBM endothelium (>70% efficiency), significantly surpassing that achieved via convection‐enhanced delivery (CED). “Sonoselective,” endothelial cell‐specific, transfection was then achieved by incorporating the CD144 promoter. Using this system, we delivered a CXCL9 plasmid to GBM endothelium. This intervention enhanced CD8^+^ effector and CD4^+^ helper T cell representation in the TME and significantly improved responsiveness of the tumor to aPD1 checkpoint inhibition. Broadly considered, this sonoselective non‐viral transfection platform may be adapted to multiple therapeutic strategies. Further, the approach holds an advantage over AAVs with tropism for brain endothelial cells due to the ability of FUS to provide locoregional targeting specifically to brain tumor endothelium.

## Graphical abstract

Delivering non‐viral, gene‐bearing nanoparticles with thiol‐decorated surfaces to gliomas with focused ultrasound yields transfection tropism for endothelial cells. From this platform, “sonoselective” transfection of glioma endothelium is achieved via incorporation of an endothelial cell‐specific promoter. Sonoselective delivery of plasmid encoding CXCL9 improves tumor immune landscape and conveys responsiveness to αPD1 immunotherapy.

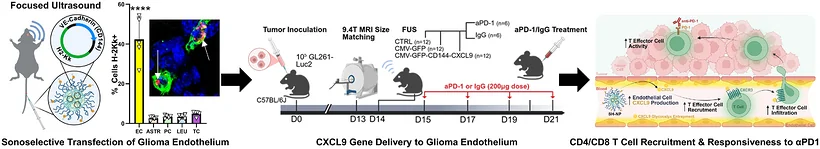

## 1 Introduction

Glioblastoma (GBM) is the most commonly diagnosed malignant tumor in the central nervous system. Prognosis for GBM patients remains poor, with a 2‐year survival rate of ∼30% and almost complete mortality [1, 2, 3]. Further, the median survival for GBM has only increased 4 months since 2005 [1, 4], highlighting the ongoing need for more innovative and effective therapies. A major challenge to treating GBM is the blood‐brain barrier (BBB), which restricts passage of approximately 98% of small molecules into the brain, reducing efficacy and necessitating high doses that increase toxicity [5, 6]. In GBM, rapid tumor growth and angiogenesis disrupt the BBB, forming a leaky blood‐tumor barrier (BTB) with pathological features like increased permeability [7, 8], drug efflux pump overexpression [9, 10], immunosuppressive signaling [11], and dysregulated protein synthesis [12, 13, 14]. While these abnormalities drive tumor progression and therapy resistance, they also present unique opportunities for targeted treatment strategies.

Nanoparticles (NPs) enable the targeted delivery of gene vectors for a wide range of therapeutic targets [15, 16, 17, 18, 19]. However, passive NP accumulation in GBM due to leaky vasculature and the so‐called “enhanced permeability and retention” (EPR) effect is highly heterogeneous within tumors and only allows a miniscule fraction of an administered dose to reach the tumor [20]. To bypass the BB/BTBs, therapeutics can be administered via convection‐enhanced delivery (CED). While this method is used clinically, it requires an invasive and risky procedure [21, 22]. Focused ultrasound (FUS) is an alternative, non‐invasive approach to enhance NP delivery, involving the intravenous (IV) administration of FDA‐approved gas‐filled microbubbles (MBs) at the time of FUS treatment [23, 24, 25]. When exposed to ultrasound waves, MBs oscillate and generate mechanical forces that temporarily disrupt vasculature, facilitating therapeutic delivery. Our group and others have demonstrated that FUS can significantly improve NP delivery across the BBB and to brain tumors [24, 25, 26, 27, 28, 29]. Beyond enhancing delivery, FUS has also been shown to decrease interstitial fluid pressure and promote convection transport in tumors, further improving NP delivery [27]. Importantly, FUS is being employed clinically to assess the safety and efficacy of repeated using FUS for therapeutic delivery in Alzheimer's and GBM patients [1, 30].

In addition to carrying diverse payloads, NPs can be functionalized with surface‐bound targeting ligands to direct them toward receptors and molecules uniquely expressed or overexpressed by target tissues [31]. For GBM, NPs are often engineered to bind transferrin receptors [32], neuropilin‐1 [33, 34], integrin receptors [35, 36], and the epidermal growth factor receptor [37]. Another underexplored target for GBM NP delivery is exofacial thiols, or free thiols present on the cell surface. Exofacial thiols are enriched within tumors due to high metabolic activity, acidic pH, increased redox activity, and dysregulated protein synthesis in the TME [38, 39, 40]. These disrupted processes can lead to protein misfolding, resulting in the exposure of free thiols that can readily form disulfide bonds with other thiol‐reactive groups. Previous studies have leveraged thiol groups for a variety of applications including polymer crosslinking [41, 42, 43], disulfide bond cleavage in oxidative environments [41, 44, 45], and the conjugation of targeting ligands or nucleic acids [44, 46, 47, 48]. However, these efforts have largely focused on improving cargo loading efficiency or enabling stimuli‐responsive cargo release, rather than exploiting thiols as a direct cellular targeting mechanism. More recent work has explored thiol reactivity in vivo, such as using lipid‐based nanoparticles functionalized with maleimide or folate to target murine hepatoblastoma [49], or employing cysteine‐modified TLR7 agonists for in situ tumor vaccination [50, 51]. Here, we present the first study to harness exofacial thiol targeting using a polymeric nanoparticle platform, and the first application of this strategy for GBM.

Polymeric NP formulations offer several advantages over viral vectors for gene delivery. While viral vectors exhibit exceptional transduction efficiency, they pose significant challenges, including risks of insertional mutagenesis, immunogenicity, high production costs, and difficulty in redosing patients [31, 52]. In contrast, NPs provide a safer, more cost‐effective alternative with greater flexibility in chemical design and scalability [2, 53, 54, 55]. Herein, we used a PEGylated‐polyethyleneimine (PEI) NP vector. While PEI has shown great promise in preclinical and clinical studies, its high positive charge can cause opsonization and cytotoxicity [56, 57]. To address these limitations, we employ dense PEGylation, a process that coats NPs with a neutrally charged polyethylene glycol (PEG) layer, which allows NPs to evade immune recognition, reduces opsonization, and enhances delivery efficiency [26, 58].

Herein, we developed a therapeutic platform to target and transfect the dysfunctional BB/BTB in tumors. Previously, our group demonstrated sonoselective transfection of the endothelium in healthy brain tissue using plasmids electrostatically bound to cationic MBs in combination with low‐pressure FUS [54]. Rather than electrostatically associating plasmids with MBs, we encapsulated plasmids within SH‐NPs to provide additional protection from immune clearance in the bloodstream. Using this approach, we established a platform for highly selective transfection of tumor endothelium, thus introducing a versatile strategy for targeting endothelial dysfunction in GBM. Notably, delivering a CXCL9‐expressing plasmid to GBM endothelium with this system beneficially remodeled the tumor immune landscape and enhanced tumor response to aPD1 immunotherapy.

## 2 Results

### 2.1 Characterization and In‐Vitro Testing of a Plasmid‐bearing Nanoparticle Designed to Target Exofacial Thiols in the Tumor Microenvironment

We first designed, characterized, and tested densely PEGylated, non‐viral, plasmid‐bearing nanoparticles (NPs) that incorporate thiol (SH) groups on their surface. SH‐NPs were approximately 49 nm in size with a slightly positive surface charge of +2 mV (Figure 1A) and polydispersity index (PDI) of 0.187 ± 0.048. The overarching concept of the SH‐NPs was to leverage exofacial thiols on cells in the tumor microenvironment [38, 40] to facilitate internalization and improve transfection (Figure 1B,C). SH‐NPs were able to transfect GL261 cells in vitro, surpassing reporter expression levels generated by transfecting cells with an equivalent amount of plasmid and Lipofectamine 3000, a commonly used transfection reagent (Figure 1D) (*38*, *39*). Because tumor endothelial cells are also an enticing target for non‐viral gene therapy, we tested whether tumor endothelial cells might also be enriched for exofacial thiols and a target for SH‐NPs. Using an Ellman's reagent assay, we compared exofacial thiols on control bEnd.3 cells cultured in normal media to those on bEnd.3 cells cultured in media from GL261‐Luc2 cells, a stimulus intended to replicate exposure of endothelial cells to soluble constituents in the tumor microenvironment (Figure 1E). Endothelial cells exposed to tumor cell conditioned media exhibited an ∼8‐fold increase in exofacial thiols (Figure 1F), consistent with their potential as a target for SH‐NPs.

**FIGURE 1:**
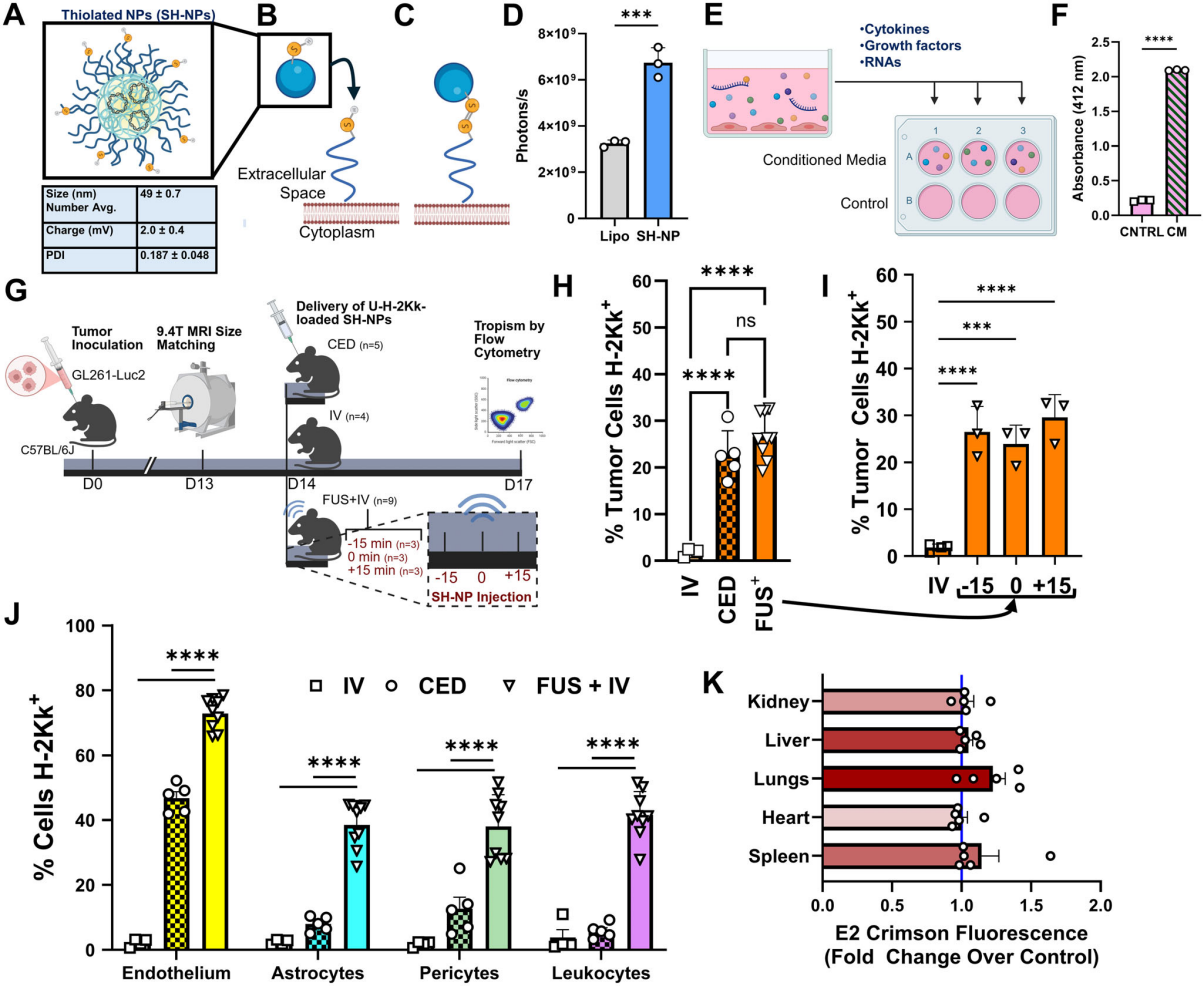
Focused ultrasound‐mediated delivery of non‐viral SH‐NPs to GL261‐Luc2 tumors yields high overall intratumor transfection and exceptional endothelial cell tropism. (A) Schematic and characterization of physiochemical properties of SH‐NPs. (B,C). Schematic of targeting free surface thiols to exofacial thiols on cells (B) to augment transfection (C). (D) Luciferase expression following transfection of GL261 cells with Lipofectamine3000 (Lipo) and SH‐NPs (Unpaired *t*‐test; ^***^ *P* < 0.001). (E) Schematic of experimental quantification of exofacial thiols on endothelial cells. Created in BioRender. Price, R. (2026). https://BioRender.com/ohurvqb. (F) Ellman's reagent analysis of exofacial thiols on control (CNTRL) bEnd.3 cells and bEnd.3 cells treated with GL261‐Luc2 tumor conditioned media (CM) (Unpaired *t*‐test; ^****^ *p* < 0.0001). (G) Timeline of experiment comparing focused ultrasound‐mediated transfection to that achieved via intravenous (IV) injection or convection‐enhanced delivery (CED). Created in BioRender. Price, R. (2026). https://BioRender.com/9oh2hur. (H) Bar graph of GL261‐Luc2 tumor cell transfection following SH‐NP delivery via IV, CED, or FUS + IV (One‐Way ANOVA with Tukey's multiple comparisons test; ^****^ *p* < 0.0001). (I) Tumor cell transfection following SH‐NP administration 15 min before (‐15), at the time of (0) or 15 min after (‐15) FUS treatment (Two‐Way ANOVA with Tukey's multiple comparisons tests; ^****^ *p* < 0.0001). (J) Bar graph of endothelial cell, astrocyte, pericyte and leukocyte transfection following SH‐NP delivery via IV, CED or FUS + IV (One‐way ANOVAs with Tukey's multiple comparisons tests; ^****^ *p* < 0.0001). K) Ex vivo imaging of off‐target organs following IV injection of E2‐Crimson plasmid‐loaded SH‐NPs (*n* = 5 per group).

### 2.2 Focused Ultrasound‐Mediated Delivery of SH‐NPs Yields Exceptional Tropism for Glioma Endothelial Cells

We next tested the ability of SH‐NPs to transfect various cell types in the tumor microenvironment. NPs can be delivered through multiple administration routes, and these routes can ultimately impact the magnitude and tropism (i.e., cell type preference) of transfection. To determine whether a particular administration route offers superior tumor cell transfection with SH‐NPs, we administered SH‐NPs loaded with a ubiquitously‐expressing H‐2Kk reporter plasmid (U‐H‐2Kk) (Figure S3) intravenously (IV; 40 µg pDNA), via convection‐enhanced delivery (CED) (20 µg pDNA), or using FUS after IV administration (FUS + IV) (Figure 1G) (40 µg pDNA). Cells were analyzed by flow cytometry using the gating strategy outlined in Figure S1. As expected, IV administration of SH‐NPs resulted in poor tumor cell transfection (1.8%). However, both CED and FUS + IV significantly improved tumor cell transfection, with comparable efficacy between the two groups (22.7% and 26.6%, respectively; Figure 1H). We also investigated whether timing of SH‐NP delivery relative to FUS treatment would impact transfection efficacy, as optimal timing of NP delivery relative to FUS treatment is not well defined for NP constructs [27, 59]. We treated mice with SH‐NPs either 15 min before (−15, *n* = 3), at the time of (0, *n* = 3), or 15 min after (+15, *n* = 3) FUS treatment and observed that within this window, timing of SH‐NP delivery did not impact tumor cell transfection (Figure 1I).

Because the delivery of SH‐NP with both CED (20 µg pDNA) and FUS + IV (40 µg pDNA) yielded nearly identical tumor cell transfection efficiencies, we were able to compare how these 2 delivery methods relatively affect tropism for other key cell types in the TME. We evaluated endothelial cells (CD31^+^), astrocytes (ASCA‐2^+^), pericytes (CD146^+^), and leukocytes (CD45^+^). Again, IV administration of SH‐NPs in the absence of FUS resulted in poor transfection across all cell types. Compared to IV, as expected, there was a significant improvement in transfection efficacy in all cell types following both CED and FUS + IV (Figure 1J). Compared to CED, FUS + IV resulted in superior tropism for endothelial cells (73% vs. 47%), astrocytes (39% vs. 8%), pericytes (38% vs. 13%), and leukocytes (42% vs. 3%). As with the tumor cells, timing of SH‐NP delivery relative to onset of FUS treatment did not impact transfection efficacy for all cell types (Figure S2). Analysis of off‐target SH‐NP transfection after IV injection of SH‐NPs loaded with an E2‐Crimson‐expressing plasmid showed off‐target transfection was negligible in the observed major organs, with some minimally detectable in the spleen and lung. E2 Crimson was detectable in the spleen in only 1 of 5 mice (Figure 1K).

### 2.3 Sonoselective Transfection of Glioma Endothelium

We reasoned that the exceptionally high tropism of FUS‐delivered SH‐NPs for tumor endothelium (Figure 1J) could be leveraged to develop a high‐efficiency, endothelial cell‐specific platform for gene therapy. To achieve endothelial cell specificity, we loaded SH‐NPs with an H‐2Kk‐encoding plasmid driven by CD144, an endothelial cell‐specific promoter (CD144‐H‐2Kk, Figure S4), instead of U‐H‐2Kk (Figure S3). Again, we used FUS + IV to deliver SH‐NPs and employed flow cytometry to assess cellular transfection (Figure 2A). By delivering SH‐NPs loaded with CD144‐H‐2Kk plasmids via FUS, we successfully achieved highly efficient sonoselective transfection of the endothelium (Figure 2B), with over 40% transfection of endothelial cells and negligible transfection in astrocytes, pericytes, leukocytes, and tumor cells (Figure 2C). Confocal imaging of cross‐sections that were immunofluorescently stained for H‐2Kk and endothelium (CD31) confirmed that, while FUS‐mediated delivery of SH‐NPs encapsulating U‐H‐2Kk transfected both endothelial and non‐endothelial cells in the TME, transfection following FUS‐mediated delivery of SH‐NPs loaded with CD144‐H‐2Kk was highly selective for endothelial cells (Figure 2D). Quantitative analysis of these cross‐sections confirmed that there was a significant increase in colocalization between H‐2Kk and CD31 with the CD144‐H‐2Kk promoter, with nearly 100% of detectable H‐2Kk staining overlapping CD31^+^ endothelium (Figure 2E). This demonstrates that loading SH‐NPs with an endothelial cell–specific promoter plasmid and delivering them via FUS + IV achieves highly sonoselective transfection.

**FIGURE 2:**
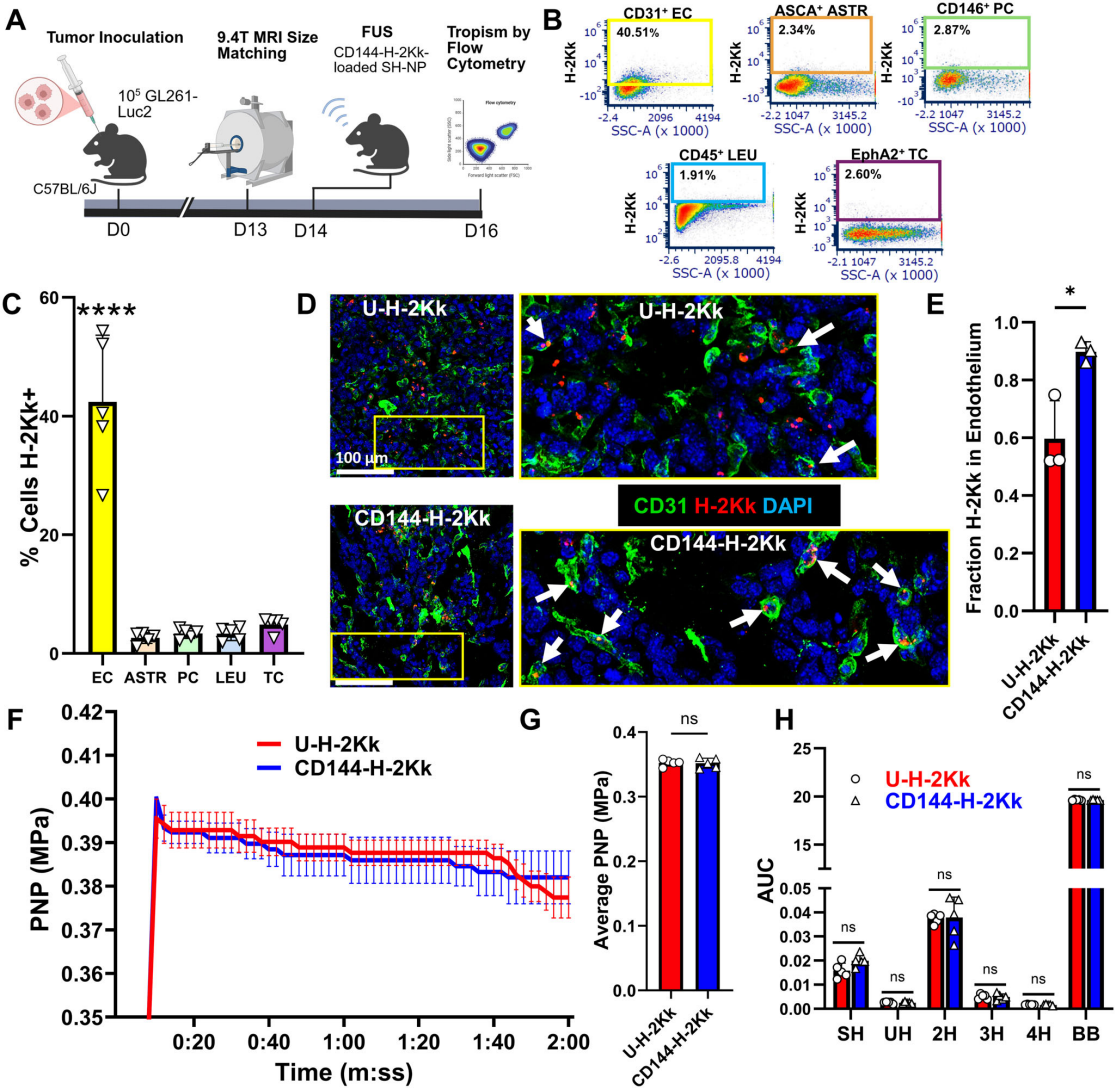
Incorporation of CD144 promoter into plasmid carried by non‐viral SH‐NPs facilitates sonoselective transfection of GL261‐Luc2 endothelium. (A) Timeline of the experiment to assess specificity and magnitude of endothelial cell transfection. Created in BioRender. Price, R. (2026). https://BioRender.com/nmksmoz. (B) Representative flow cytometry plots for H‐2Kk transfected cells in the tumor microenvironment following FUS + IV delivery of CD144‐H‐2Kk plasmid‐loaded SH‐NPs. (C) Bar graph of transfected cells in the tumor microenvironment (One‐way ANOVA followed by Dunnett's multiple comparisons tests versus endothelial cells; ^****^ *p* < 0.0001 vs. all other groups). (D) Confocal images of GL261‐Luc2 tumors following FUS‐mediated delivery of SH‐NPs bearing U‐H‐2Kk (top) and CD144‐H‐2Kk plasmids (bottom). Scale bars = 100 µm. Arrows denote colocalization of H‐2Kk and CD31. Note that H‐2Kk is present in tumor parenchyma with the H‐2Kk promoter, but not with CD144. (E) Bar graph of co‐localization of H‐2Kk and CD31 expression (Unpaired *t*‐test; ^*^ *p* < 0.05). (F) Applied peak‐negative pressure (PNP) over time during passive cavitation detection (PCD)‐modulated FUS treatments. Each line represents the average applied PNP over 3 sonication points per 5 mice ± SEM. (G) Average PNP over 3 sonication points per mouse (Unpaired t‐test). (H) Bar graph of subharmonic (SH), ultraharmonic (UH), second harmonic (2H), third harmonic (3H), fourth harmonic (4H), and broadband (BB) emissions. Unpaired *t*‐tests.

For all FUS treatments performed in this study, we administered a clinically relevant FUS technique using passive cavitation detection (PCD) to provide real‐time acoustic monitoring and control of treatments, ensuring safety [60, 61]. FUS treatments delivering both U‐H‐2Kk and CD144‐H‐2Kk plasmids in SH‐NPs resulted in similar applied peak negative pressure (PNP) patterns over the course of treatments (Figure 2F), with an average applied PNP of ∼0.35 MPa for both groups (Figure 2G). Consistent with this result, key acoustic emission signatures were also similar between groups (Figure 2H). Because both mechanical energy deposition and MB response during U‐H‐2Kk and CD144‐H‐2Kk delivery treatments were unchanged, differences in H‐2Kk expression tropism may be attributed to incorporation of the CD144 promoter and not differences in FUS treatments.

### 2.4 Improved Immune Cell Recruitment Using Sonoselective Transfection of Chemokines

One therapeutic application of the sonoselective gene delivery platform is to deliver chemokine plasmid(s) to the endothelium to augment the recruitment of CD8 effector and CD4 helper T cells, as well as NK cells, to the TME (Figure 3A). To this end, we designed and generated a plasmid wherein the CD144 promoter drives expression of CXCL9, a chemokine that recruits T cells and NK cells via signaling to the CXCR3 receptor (Figure 3B). The plasmid also contained a GFP reporter driven by CMV. Using flow cytometry and following the gating strategy outlined in Figure S5, we confirmed that this plasmid elicited enhanced CXCL9 expression in transfected bEnd.3 cells (Figure 3C). Using a co‐culture assay with immortalized cytotoxic T cells and CXCL9‐transfected bEnd.3 cells, as well as flow cytometry with the gating strategy outlined in Figure S6, we also verified that bEnd.3 cells transfected with this CXCL9 plasmid exhibit augmented T cell recruitment (Figure 3D).

**FIGURE 3:**
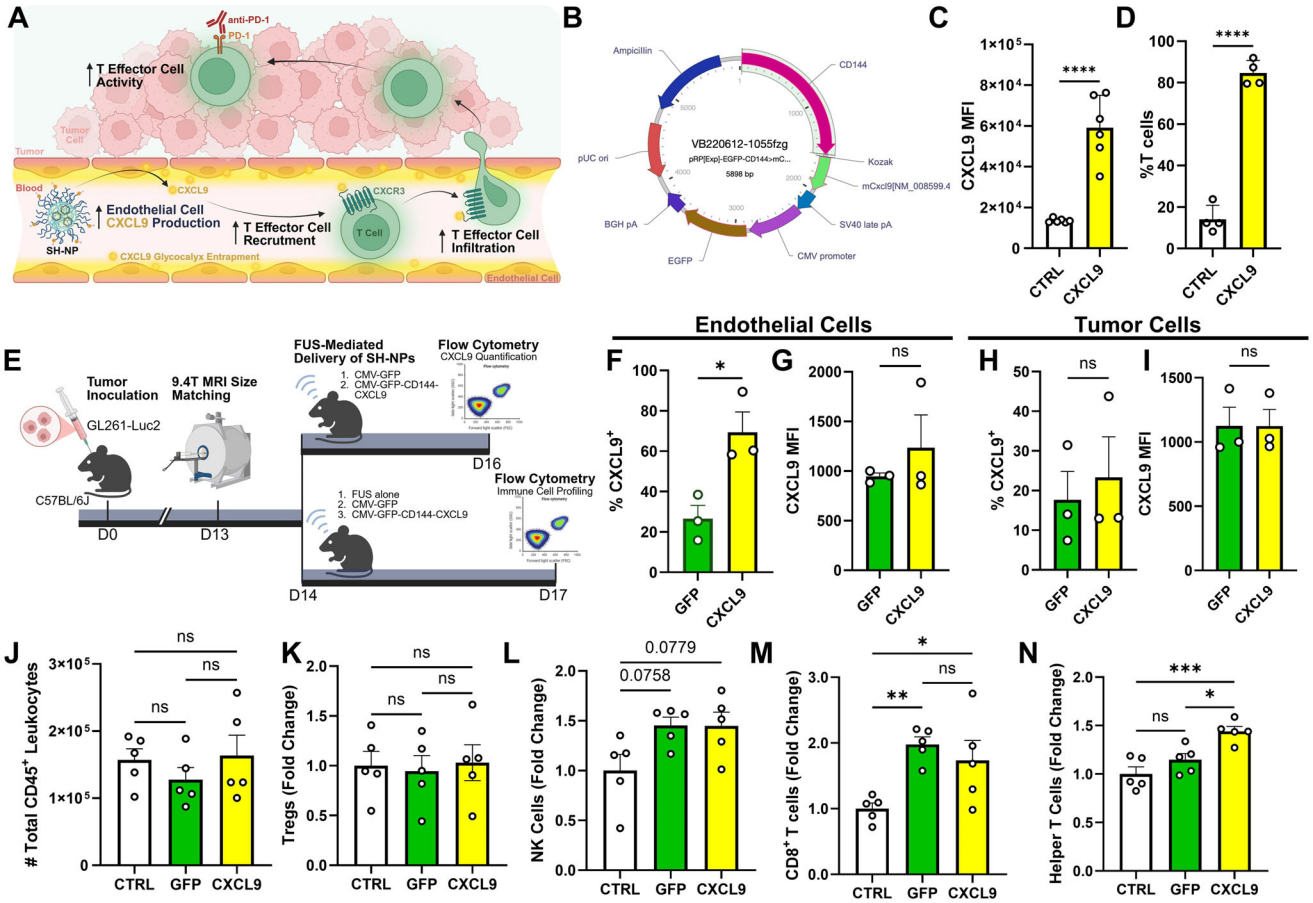
Sonoselective transfection of GL261‐Luc2 tumor endothelium with CD144‐CXCL9 plasmid beneficially remodels tumor immune landscape. (A) Schematic demonstrating how FUS‐mediated, endothelial‐cell specific, CXCL9 transfection with SH‐NPs may augment CXCR3^+^ T cell recruitment into brain tumors. Created in BioRender. Price, R. (2026). https://BioRender.com/cgw0tl5. (B) Design of therapeutic plasmid. CD144 drives the expression of CXCL9, while CMV drives GFP expression. Control plasmid lacks CD144‐CXCL9 component. (C) CXCL9 expression in bEnd.3 cells, transfected with plasmid in panel B, compared to untreated bEnd.3 cells (CTRL). (Unpaired *t*‐test; ^****^ *p* < 0.0001). (D) T cell recruitment following co‐culture with bEnd.3 cells transfected with CXCL9 plasmid. (Unpaired *t*‐test; ^****^ *P* < 0.0001). (E) Timeline of experiment to measure CXCL9 expression in endothelium and profile intratumor immune cell representation after FUS‐mediated delivery of SH‐NPs carrying either CD144‐CXCL9 or control plasmid (GFP). Created in BioRender. Price, R. (2026). https://BioRender.com/a357czb. (F) Percent of endothelial cells expressing CXCL9 (Unpaired *t* test; *p*<0.05). G) MFI of CXCL9^+^ endothelial cells. (Unpaired t test). H) Percent of tumor cells expressing CXCL9 (Unpaired *t*‐test). (I) MFI of CXCL9^+^ tumor cells. (Unpaired *t*‐test). (J–N) Bar graphs of intratumor immune cell representation: Total CD45+ leukocytes (J) and fold changes over untreated control (CTRL) for Tregs (K), Natural Killer (NK) cells (L), CD8^+^ Effector T cells (M), and CD4^+^ Helper T Cells (N). (One‐Way ANOVAs with Tukey's multiple comparisons tests. ^*^ *p* < 0.05, ^**^ *p* < 0.01, ^***^ *p* < 0.001).

We then tested whether the sonoselective transfection of GL261‐Luc2 endothelium with CXCL9 plasmid in vivo would yield an increase in chemokine expression. We treated two groups of GL261‐Luc2 tumor‐bearing mice: (1) to account for any stimulation for the reporter plasmid we treated mice with GFP plasmids driven by CMV after FUS + IV (CMV‐GFP) and (2) our therapeutic plasmid administered via FUS + IV (CMV‐GFP‐CD144‐CXCL9, termed “CD144‐CXCL9”; Figure 3E). Using flow cytometry and the gating strategy outlined in Figure S7, we observed that delivery of the CD144‐CXCL9 plasmid generated an ∼2.5‐fold increase in the percentage of CXCL9 expressing cells (Figure 3F), although there was no detectable difference in CXCL9 MFI among CXCL9 expressing cells (Figure 3G). As expected, since CXCL9 was driven by an endothelial specific promoter, we observed no increase in CXCL9 expression in tumor cells (Figure 3H,I).

Next, we investigated the impact of increased endothelial cell CXCL9 expression on immune cell recruitment to the TME. We treated three groups of GL261‐Luc2 tumor‐bearing mice: (1) control (CTRL) mice receiving no additional treatments, (2) mice receiving SH‐NPs loaded with CMV‐GFP after FUS + IV to account for any FUS‐ and/or GFP‐induced immune recruitment, and (3) mice treated with SH‐NPs loaded our CXCL9 plasmids after FUS + IV (Figure 3E). Three days after treatment, tumor‐bearing brain quadrants were harvested and analyzed by flow cytometry, following the gating strategy outlined in Figure S8, to profile the representation of NK cells (CD45^+^ CD3^−^ NK1.1^+^), cytotoxic T cells (CD45^+^ CD3^+^ CD8^+^), Tregs (CD45^+^ CD3^+^ CD4^+^ FOXP3^+^), and helper T cells (CD45^+^ CD3^+^ CD4^+^ FoxP3^−^) in the TME. We observed no differences between the 3 groups in both the total number of CD45^+^ leukocytes (Figure 3J) and the proportion of leukocytes that were immunosuppressive Tregs (Figure 3K). There were strong trends (P = ∼0.08) toward increased NK cell representation in both the GFP and CXCL9 plasmid groups (Figure 3L). Sonoselective delivery of SH‐NPs carrying both CD144‐CXCL9 and CMV‐GFP plasmids yielded similar significant increases in CD8^+^ cytotoxic T cell infiltration compared to controls (1.9‐ and 1.7‐fold increase, respectively; Figure 3M). Moreover, sonoselective transfection of GL261‐Luc2 endothelium with our CXCL9 plasmid increased CD4^+^ helper T cells by 1.5‐fold compared to controls (Figure 3N).

### 2.5 Immunotherapeutic Responses to Sonoselective Transfection of GL261‐Luc2 Tumors With CXCL9 Plasmid

After confirming that sonoselective transfection of GL261‐Luc2 endothelium with the CXCL9 plasmid beneficially remodels tumor immune landscape by augmenting the representation of CXCR3^+^ immune cells in the TME, we tested whether this intervention could control tumor growth, both with and without the addition of aPD1 checkpoint inhibition (Figure 3A). To do this, we treated three groups of mice as before: (1) control (CTRL) mice receiving no additional treatments, (2) mice receiving SH‐NPs loaded with CMV‐GFP after FUS + IV, and (3) mice treated with SH‐NPs loaded our CXCL9 plasmids after FUS + IV. We then treated mice within groups 2 and 3 with either 200 ug of aPD1 or IgG control every two days for a total of four treatments (Figure 4A). Mice were observed until they exhibited behaviors consistent with humane endpoint criteria. To isolate the influence of endothelial cell‐specific CXCL9 expression, we first compared the GFP+FUS+IgG group to the CXCL9+FUS+IgG group. While there was no difference in animal survival (Figure 4B), one of the CXCL9+FUS+IgG mice demonstrated complete tumor eradication, which was confirmed by the absence of luciferase signal upon bioluminescence imaging (Figure 4C). This mouse was euthanized on day 62, followed by immunofluorescence imaging to determine whether resident memory T cells (CD103^+^ CD69^+^) were now present. The brain region where the tumor resided was evident by hypercellularity (Figure 4D). This region was characterized by high levels of CD45 expression, much of which was co‐localized with both CD103 and CD69 staining (Figure 4E), consistent with the presence of resident memory T cells. In contrast, CD103 and CD69 expression was almost entirely absent on the contralateral side of the brain (Figure 4F,G). Finally, we compared mouse survival in the untreated control (CTRL) group, GFP+FUS+aPD1, and CXCL9+FUS+PD1 groups. Mice in the CXCL9+FUS+aPD1 group survived significantly longer (median = 28 days) than mice in the CTRL group (median = 21.5 days). GFP+FUS+aPD1 (median = 23 days) and CTRL were not different from each other (Figure 4H). This indicates that immune landscape remodeling in response to sonoselective CXCL9 delivery can indeed potentiate checkpoint inhibition in a model wherein aPD1 is relatively ineffective.

**FIGURE 4:**
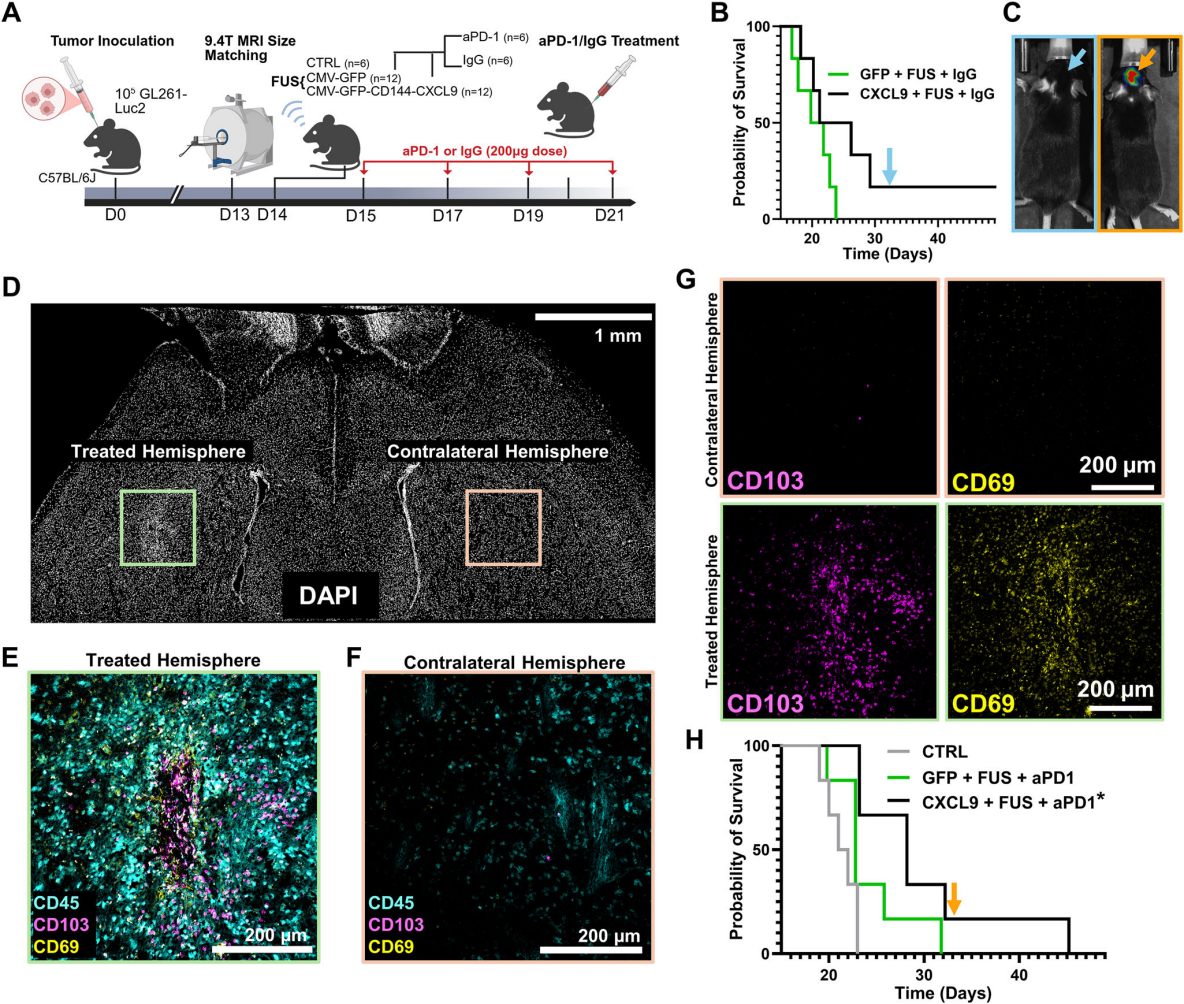
Immunotherapeutic responses to the sonoselective transfection of GL261‐Luc2 tumor endothelium with CD144‐CXCL9 plasmid. (A) Timeline of the experiment to assess the impact of CD144‐CXCL9 plasmid‐bearing SH‐NP delivery with FUS in combination with aPD1 checkpoint inhibition. Created in BioRender. Price, R. (2026). https://BioRender.com/0b2yr7c. (B) Kaplan‐Meier curve comparing CXCL9 plasmid delivery to control GFP plasmid delivery. There were no differences between groups, but the GL261‐Luc2 tumor was eradicated in one CXCL9+FUS+IgG treated mouse. Mantel‐Cox regression test. (C) Absence of bioluminescence imaging confirms tumor eradication (cyan arrow) in a CXCL9+FUS+IgG group mouse. A tumor‐bearing mouse from the CXCL9+FUS+aPD1 group in panel H, imaged at the same timepoint (day 33), is provided as a positive control for bioluminescence signal (orange arrow). (D) Low‐magnification confocal image of DAPI staining in CXCL9+FUS+IgG mouse exhibiting tumor eradication. Note hypercellularity in the region of the treated hemisphere where tumor was located. (E,F) Higher magnification confocal images of CD45, CD103, and CD69 expression at the site of tumor eradication (E) and in the contralateral hemisphere (F). Cells expressing all 3 proteins appear white and primarily reside in the middle of the eradication site. (G) Single‐channel confocal images of CD103 and CD69 expression in treated and contralateral hemispheres. H) Kaplan‐Meier curve comparing survival of untreated control (CTRL) mice to mice treated with aPD1 subsequent to FUS‐mediated delivery of SH‐NPs carrying either GFP control plasmid (GFP+FUS+aPD1) or CD144‐CXCL9 plasmid (CXCL9+FUS+aPD1). Mantel‐Cox regression test. ^*^ *p* < 0.05 vs. CTRL.

## 3 Discussion

GBM remains incredibly difficult to treat, with standard of care therapies yielding a median survival of only 15 months. Dysfunction of the GBM endothelium promotes disease progression and treatment resistance through multiple mechanisms. For example, dysfunctional endothelial cells have a limited capacity to recruit beneficial NK and T cells from the bloodstream, contributing to an immunosuppressive TME. Because dysfunctional GBM endothelium is largely implicated in disease progression, it is also an attractive and powerful target for gene therapy interventions. In this study, we developed a non‐viral, gene‐bearing, polymer nanoparticle that exhibits remarkably high tropism for GBM endothelium when delivered with FUS. Incorporating an endothelial cell‐specific promoter into this system yielded highly efficient, endothelial cell‐specific, sonoselective transfection. As an exemplar therapeutic application of this system to the GBM endothelium, we delivered the plasmid for a chemokine (CXCL9) capable of recruiting CXCR3^+^ immune cells to the TME. This treatment (i) stimulated beneficial remodeling of the tumor immune landscape via increased representation of CD8^+^ effector and CD4^+^ helper T cells, (ii) showed the potential to eradicate tumors and generate tissue‐resident memory T cells, (iii) and conveyed responsiveness to aPD1 in a tumor model that is otherwise refractory to checkpoint inhibition. The overall ability of this strategy to spatially target endothelial cell‐specific gene delivery to brain tumors with FUS represents a distinct advantage over AAV‐based strategies that exhibit high tropism for brain endothelial cells, but do not offer spatial control within the brain. Moreover, its possible that this platform may be transferred to other solid tumor indications, providing a versatile tool for gene therapy screening across multiple cancer types.

Cells within the TME are enriched with exofacial thiols due to dysregulated protein synthesis, increased metabolic activity, and the acidic pH, all of which are characteristic of tumors [4, 8, 38, 50, 51]. In an effort to exploit this unique pathological hallmark, we engineered SH‐NPs to contain surface‐bound thiol groups. Free thiols are highly reactive and form strong covalent bonds with other thiol groups [40], like exofacial thiols, which, in the context of NP delivery, can facilitate cellular uptake and internalization. While our results are consistent with the potential for SH group decoration to augment tumor endothelial cell transfection, and exofacial thiols are known to enhance cell internalization via receptor‐mediated endocytosis [38], it is important to note that this was not empirically verified. Yet, given the impact of SH decoration on nanoparticle transport, corona formation, and circulation time, as well as the potential for interaction effects with FUS, in vivo empirical comparisons to simpler PEG‐coated nanoparticles would still not clearly delineate a mechanistic role for SH‐group decoration in FUS‐mediated transfection augmentation. SH‐NPs also possess favorable physicochemical properties, including a small size and neutral charge, which enable them to pass through dense, highly negatively charged extracellular matrix in brain tissue [58].

We used flow cytometry to characterize the transfection tropism of SH‐NPs across various cell types in the TME to better inform therapeutic payloads for future studies. While in vivo transfection is typically analyzed by immunofluorescence or in/ex vivo fluorescence imaging [62, 63], these methods are limited in their ability to quantify transfection efficiency across multiple cell types and lack cellular resolution, respectively. Flow cytometry offers a powerful alternative that can be used to analyze individual cells, providing a comprehensive understanding of a gene delivery platform and its transfection capabilities. In this study, SH‐NPs were loaded with a U‐H‐2Kk reporter plasmid, and flow cytometry was used to quantify the percentage of transfected cells, providing insights into the number of successfully treated cells.

NPs for GBM therapy can be administered via several different routes, each with distinct advantages and limitations [64]. As such, we evaluated the efficacy of SH‐NPs delivered via IV, CED, and IV + FUS. As expected, IV administration alone resulted in poor transfection due to its reliance on the EPR effect, which can be inefficient, even for targeted formulations [7, 8]. While CED improved transfection, FUS + IV significantly outperformed CED in all cell types other than tumor cells, suggesting an unexpected synergy between SH‐NPs and FUS. This may be due to FUS‐mediated upregulation of cellular uptake mechanisms, such as transcytosis and endocytosis, as well as increased vascular permeability from tight junction disruption [60]. Additionally, some studies demonstrate that higher‐pressure FUS can modulate the presence of free thiols [65, 66, 67].

We also evaluated whether the timing of SH‐NP administration relative to FUS influenced transfection efficacy, as optimal timing for NP delivery relative to FUS treatment remains poorly characterized and is likely dependent on individual NP formulations [27, 59]. SH‐NPs were administered either 15 min before, at the time of, or 15 min after FUS treatment. No significant differences in transfection efficiency or MFI were observed within this 30‐min timeframe. This characterization of SH‐NP transfection provides insights for optimizing experimental timelines and informing the design of future therapeutic strategies. Of note, interactions between the SH‐NPs and serum proteins were not studied, and colloidal stability of the SH‐NPs in serum was not assessed. Given the potential importance of such interactions in dictating NP behavior in vivo, such studies are warranted for optimizing the therapeutic strategy going forward.

One noteworthy observation is that ∼73% of endothelial cells were successfully transfected when SH‐NPs carrying U‐H‐2Kk reporter plasmid were delivered with FUS. Moreover, endothelial cells transfected via FUS + IV exhibited the highest MFI, indicating superior transgene expression per cell. While non‐viral delivery vectors are generally less efficient than viral vectors, our approach achieved transfection efficiencies comparable to adeno‐associated viruses (AAVs) [68, 69]. This highlights the potential of this gene delivery strategy, especially since the non‐viral component largely obviates concerns about cost and immunogenicity that are commonly associated with AAVs. Additionally, despite their nature to phagocytose foreign material, about 43% of leukocytes (CD45^+^ cells) were transfected following FUS + IV. Immune cells within the TME are exposed to oxidative stress that may increase the presence of exofacial thiols and facilitate immune cell transfection. Our results also demonstrate the versatility of therapeutic platforms for FUS‐mediated delivery of SH‐NPs to screen different therapies for GBM.

To further improve tropism for endothelial cells, we loaded SH‐NPs with an H‐2Kk reporter plasmid driven by CD144, an endothelial‐specific promoter. Using flow cytometry, we profiled transfection across cells within the TME and found efficient transfection specifically in the endothelium, with negligible transfection of other cell types. Notably, when using the CD144‐H‐2Kk reporter plasmid, we observed transfection in 42% of endothelial cells, compared to 73% using the U‐H‐2Kk plasmid. As FUS energy deposition and MB activity were comparable between treatments, this difference can be attributed to the use of the CD144 promoter, which may not drive gene expression as efficiently as the H‐2Kk promoter. Further optimization of FUS treatment parameters may enhance transfection efficiency. Nevertheless, by leveraging FUS to deliver CD144‐H‐2Kk‐loaded SH‐NPs, we achieved highly specific, or sonoselective, transfection of endothelial cells, demonstrating the potential of this platform to precisely and efficiently target the dysregulated endothelium within the TME.

We next sought to evaluate the therapeutic potential of this platform in GBM. Others have shown that engineered nanosystems can be immunotherapeutic for GBM [70], providing rationale for deploying our sonoselective transfection strategy in an immunotherapeutic capacity. In GBM, T and NK cell recruitment to the TME is impaired because endothelial cells are “anergic” [71, 72, 73], a dysregulated state wherein they transform into a non‐adhesive barrier for T and NK cell capture [73]. One potential strategy to overcome this barrier is modulation of chemokine expression to enhance recruitment of antitumor immune cells. The CXCL9 chemokine is expressed in the brain and promotes recruitment of CXCR3^+^ immune cells [74], including NK cells and T cells. However, the overall role of CXCL9 in tumor progression is complex, as CXCL9 secreted by tumor cells has been implicated in tumorigenesis [75]. Thus, we reasoned that sonoselective transfection could be therapeutic via the spatially precise targeting of CXCL9 to tumor endothelium, without enhancing CXCL9 expression in tumor cells. Indeed, sonoselective transfection of the endothelium using CXCL9 plasmid‐loaded SH‐NPs increased endothelial cell CXCL9 expression without affecting tumor cell expression. Further, this treatment resulted in an ∼1.4‐fold increase in CD4^+^ helper T cells, which is comparable to the increase achieved after AAV6‐mediated CXCL9 transduction in this same GL261 glioma model [76]. Importantly, the population of immunosuppressive Tregs remained unchanged with treatment. We also observed an increase in CD8^+^ cytotoxic T cells following delivery of both GFP and CXCL9 plasmids. One hypothesis for why SH‐NPs bearing the GFP reporter plasmid may modulate the immunosuppressive TME is through alteration of redox dynamics. Reactive oxygen species (ROS), which are elevated in tumors, can impair the activity of NK and T cells [77], contributing to immune suppression. In response to elevated ROS levels, tumors upregulate glutathione [77], a natural antioxidant that neutralizes ROS via its free thiol group. The surface of SH‐NPs is rich in free thiols and, as such, may mimic this antioxidant function by reducing ROS levels and thereby improving immune function, which could be reflected in an increase in CD8 T cell numbers. Nonetheless, while further investigation of this hypothesis is warranted, and optimization of sonoselective CXCL9 delivery magnitude via modulation of SH‐NP dosage, FUS parameters, and number of treatments represents an important future direction, the strategy used here yielded increases in both CD8 effector and CD4 helper T cells.

Finally, while sonoselective CXCL9 transfection alone did not significantly improve overall survival, one mouse did exhibit complete tumor eradication accompanied by evidence of resident memory T cells in the treated region. Furthermore, we hypothesized that the ability of sonoselective endothelial delivery of CXCL9 to augment CD4 helper and CD8 effector T cells in the TME would enhance responsiveness to aPD1. Consistent with this hypothesis, treatment with aPD1 following CXCL9 plasmid delivery to tumor endothelium resulted in a significant improvement in survival. Given these results, going forward, there may also be significant potential for sonoselective transfection of brain tumor endothelium to drive the recruitment of adoptively transferred T cell therapies to the TME.

## 4 Methods

### 4.1 SH‐NP Formulation and Characterization

Polymers used for SH‐NP formulation were (1) PEI‐g‐PEG‐SH graft copolymer of 25 kDa branched PEI modified with a 20% substitution ratio of 5 kDa SH‐PEG (Nanosoft Polymers) and (2) 25 kDa branched PEI (ThermoFisher). Polymers solutions were prepared at a PEI‐g‐PEG‐SH:PEI molar ratio of 3. The polymer solution was incubated with tris(2‐carboxyethyl)phosphine (TCEP) and a 10:1 TCEP:thiol ratio for 10 min to reduce disulfide bonds within the polymer network. SH‐NPs were then formed by the drop‐wise addition of 10 volumes of pDNA diluted in water to 1 volume of polymer solution (while vortexing) at an optimized nitrogen to phosphate (N/P) ratio of 6. The SH‐NP solution was then purged with decafluorobutane (DFB) gas to remove oxygen and filtered through Amicon Ultra Centrifugal Filters (100,000 MWCO, Sigma–Aldrich) to remove free polymer and TCEP. Prior to spinning, the empty headspace in the filters was filled with DFB gas to prevent reformation of disulfide bonds. SH‐NPs were concentrated to 1 mg/mL using DFB‐purged water, stored in inert conditions, and used the same day. The hydrodynamic diameter and polydispersity index of SH‐NPs were measured using dynamic light scattering, and ζ‐potential was measured using laser Doppler anemometry using a Zetasizer Nano ZS90 (Malvern Instruments).

### 4.2 Maintenance of Cell Lines

Research resource identifiers (RRIDs) are provided for all reagents that have them. bEnd.3 cells (ATCC, Catalog #: CRL‐2299, RRID: CVCL_0170) were maintained in high glucose DMEM (Gibco, Catalog #: 11‐965‐118) supplemented with 10% FBS (Gibco, Catalog #: A5256801) and 1 mm sodium pyruvate (Gibco, Catalog #: 11360070). Unless otherwise specified, GL261‐Luc2 cells (gifted from Dr. Pavlos Anastasiadis at the University of Maryland School of Medicine, RRID: CVCL_X986) were maintained in high glucose DMEM (Gibco) supplemented with 10% FBS, 1 mm sodium pyruvate (Gibco, Catalog #: 11360070), 1 mm non‐essential amino acids (Gibco, Catalog #: 11140050), and 100 ug/mL geneticin (Gibco, Catalog #: 10131035). GL261 cells (gifted from Dr. Pavlos Anastasiadis at the University of Maryland School of Medicine, RRID: CVCL_Y003) were maintained in high‐glucose DMEM supplemented with 10% FBS, 1 mm sodium pyruvate, and 1 mm non‐essential amino acids. Immortalized cytotoxic T cells (ATCC, Catalog #: CTLL‐2, RRID: CVCL_0227) were maintained in RPMI‐1640 Medium (Gibco, Catalog #: 11875093) supplemented with 2 mM L‐glutamine (Gibco, Catalog#: 25030081), 1 mm sodium pyruvate (Gibco, Catalog #: 11360070), 10% FBS (Gibco) and 10% T‐STIM with Con A (Becton Dickinson, Catalog #: 354115).

### 4.3 SH‐NP Transfection In Vitro

GL261 cells were seeded in 6‐well plates at a density of 1 × 10^5^ cells per well and incubated overnight to allow cell attachment. The following day, the media was replaced with 2 mL of fresh culture media, and cells were treated with 2.5 µg of luciferase plasmid DNA (Addgene, Plasmid #18964, RRID: Addgene_18964) encapsulated in SH‐NPs or in Lipofectamine3000 (Thermo Fisher, Catalog #: L3000015), according to the manufacturer. After 8 h, the media was again replaced with fresh culture media. Two days after transfection, the media was exchanged for GL261 media containing 0.5 mm D‐luciferin (GoldBio, Catalog #: eLUCK‐100). Following a 10‐min incubation, bioluminescence imaging was performed using a LagoX imaging system (Spectral Instruments, Catalog #: A1666) to evaluate luciferase expression.

### 4.4 In Vitro Free Thiol Quantification

Exofacial thiols on bEnd.3 cells were quantified using Ellman's reagent. bEnd.3 cells were maintained in high glucose DMEM (Gibco) supplemented with 10% FBS (Gibco) and 1 mm sodium pyruvate (Gibco). For media‐stimulated cells, GL261‐Luc2 cells were first cultured in DMEM (Gibco) supplemented with 10% FBS, 1 mm sodium pyruvate (Gibco), and 1 mm non‐essential amino acids (Gibco). Conditioned media was collected after 2 days. The harvested media was centrifuged at 1200 RPM to remove cellular debris, transferred onto bEnd.3 cells, and the cells were cultured for an additional 2 days. Free exofacial thiols were quantified using Ellman's reagent according to the manufacturer (ThermoFIsher Scientific, Catalog #: 22582). Briefly, 5 × 10^5^ cells were harvested, resuspended in 250 µL of PBS, and incubated with 2.5 mL reaction buffer and 50 µL Ellman's reagent for 15 min at room temperature. A spectrophotometer was then used to measure sample absorbance at 412 nm.

### 4.5 Quantification of CXCL9 Expression In Vivo

bEnd.3 cells were seeded in 6‐well plates at a density of 1 × 10^5^ cells per well and transfected with a CXCL9‐expressing plasmid (Vectorbuilder, Catalog #: VB220612‐1055fzg) using Lipofectamine3000 according to the manufacturer's instructions. Four days later, cells were analyzed using flow cytometry to assess chemokine expression. Twenty‐four hours and 12 h prior to analysis, cells were treated with 500 U/mL recombinant mouse IFN‐ γ (R&D Systems, Catalog #: 485‐MI) and 1x brefeldin‐A (Thermo Fisher Scientific, Catalog #: 00‐4506‐51), respectively. Cells were harvested and stained with Live/Dead Fixable Blue dye (1:1000, ThermoFisher Scientific, Catalog #: L23105), washed, and incubated with FcBlock [0.1 µg/mL 2.4G2 Ab (BD Biosciences, Calatog #: 553142) and 0.1% rat gamma globulin (Thermo Fisher Scientific Catalog #: PI31885 in FACS buffer: 1X PBS, 0.2% BSA (Thermo Fisher Scientific, Catalog #: BP1600‐100), and 2 mm EDTA (Thermo Fisher Scientific, Catalog #: AM9260G)]. After washing again, cells were permeabilized using 90% ice‐cold methanol for 10 min. Next, cells were stained with aCXCL9 (1:200, Biolegend, 515606, RRID: AB_1877135), washed again, and resuspended for analysis. Data were acquired using the 5‐laser Aurora Borealis spectral cytometer (Cytek, Catalog #: N7‐00003) and analysis was performed using the FCS Express software (De Novo Software, RRID: SCR_016431).

### 4.6 T Cell Co‐Culture Assay

bEnd.3 cells were transfected with CXCL9 as described above. Two days after transfection, cells were harvested and seeded at a density of 1 × 10^5^ cells per well in a 24 mm Transwell (Corning, Catalog #: 3412) plate. The following day, 500 µL of CTLL‐2 cells were pipetted into the 3.0‐µm pore polyester membrane inserts at a concentration of 2.5 × 10^5^ cells/mL per insert. Plates were incubated for 90 min at 37°C. Following incubation, media containing recruited CTLL‐2 cells and trypsinized bEnd.3 cells were collected and prepared for flow cytometric analysis. Cells were washed and stained with Live/Dead Fixable Blue dye (1:1000, Thermo Fisher Scientific, Catalog #: L23105). After washing, cells were then incubated with FcBlock and washed again. Next, cells were stained with aCD31 (1:100, Thermo Fisher Scientific, Catalog #: RM5228) and aCD45 (1:100, Thermo Fisher Scientific, Catalog #: A15395, RRID: AB_2534409), washed, and resuspended for analysis. Data were acquired using the 5‐laser Aurora Borealis spectral cytometer, and analysis was performed using the FCS Express software.

### 4.7 GL261‐Luc2 Intracranial Inoculations

Animal experiments were approved by the University of Virginia Animal Care and Use Committee (Protocol #3818), adhered to ARRIVE guidelines, and conformed to the National Institutes of Health guidelines for the use of animals in research. C57BL/6J mice (Jackson Laboratory, RRID: IMSR_JAX:000664) were housed under standard laboratory conditions (22°C and 12 h/12 h light/dark cycle) and treated between 8 and 10 weeks of age. Thawed GL261‐Luc2 cells were cultured for up to three passages. Cells (1 × 10^5^ cells per 2 µL) were resuspended in sterile 1x PBS and implanted into the right striatum of mice placed on a stereotactic frame using a syringe pump at a controlled rate of 0.5 µL/min. Cells were injected 2.0 mm lateral from the sagittal suture, 0.5 mm anterior of bregma, and 3 mm below the dura.

### 4.8 Tumor Size Matching

Prior to treatment, tumor volumes were acquired for size matching. MR images of mouse brains were obtained using a 9.4T Bruker MRI (RRID: SCR_018054). Mice were anesthetized and were retro‐orbitally injected with a gadolinium contrast agent (Multihance, Bracco Diagnostics) at a dose of 0.01 nmol diluted in saline (0.2 um) prior to T1‐weighted image acquisition. Tumor volumes were measured using a DICOM viewer (Horos Project).

### 4.9 IV Administration of SH‐NPs

Fourteen days after inoculation, size matched tumors were treated with SH‐NPs. Mice were anesthetized and injected with SH‐NPs (40 µg pDNA) intravenously.

### 4.10 CED of SH‐NPs

Fourteen days after inoculation, size matched tumors were treated with SH‐NPs. Anesthetized mice were placed on a stereotactic frame. Using the pre‐existing burr hole as a guide, a dose of an SH‐NP solution was injected directly into the tumor 3 mm below the dura using a syringe micropump at a controlled rate of 0.5 uL/min, administering 20 µg pDNA.

### 4.11 FUS‐Mediated Delivery of SH‐NPs

Fourteen days after tumor inoculation, mice were anesthetized, and size‐matched tumors were treated with FUS to enhance delivery of SH‐NPs administered via tail vein injection. Mouse heads were shaved and depilated prior to placement on the stereotactic frame of the RK‐50 FUS system (FUS Instruments) and coupled to a degassed water bath with ultrasound gel. Treatments were performed using a 1.1 MHz single‐element transducer with 3 sonication points per tumor (burst length: 10 ms, burst period: 2000 ms, number of sonications: 60). Real‐time passive cavitation detection (PCD) monitoring was used to modulate sonication pressures. Parameters for this system included a starting pressure of 0.2 MPa, a maximum pressure of 0.4 MPa, pressure increments of 0.05 MPa, a pressure drop of 5% when the emission threshold was exceeded, 20 baseline sonications without microbubbles, AUC bandwidth of 500 Hz, AUC standard deviations of 10, and frequency selection of the first and second ultra‐harmonic and the subharmonic. For FUS treatments, mice were injected intravenously with albumin‐shelled microbubbles (Optison) at a dose of 3 × 10^5^ microbubbles/g immediately at the start of FUS, and SH‐NPs were administered at a dose of 40 µg pDNA either 15 min before (−15) at the time of (0) or 15 min after (+15) the start of FUS treatment (FUS + IV).

### 4.12 PCD Analysis

Acoustic emissions data were collected with a hydrophone built into the center of the FUS transducer, and data were processed using a custom MATLAB script (MathWorks, RRID: SCR_001622. The area under the curve of the acoustic emissions was calculated at the subharmonic and ultra‐harmonics after applying a 300 Hz bandwidth filter. Broadband emissions were calculated by summing acoustic emissions and removing emissions at the fundamental frequency, harmonics, subharmonics, and ultra‐harmonics.

### 4.13 Flow Cytometric Analysis of H‐2Kk Transfection Efficacy

Prior to treatment, SH‐NPs were loaded either a U‐H‐2Kk plasmid (Miltenyi Biotec, Catalog #: 30‐092‐083) or CD144‐H‐2Kk plasmid (VectorBuilder, Plasmid #: VB240821‐1392jvd). To analyze cellular tropism, mice were euthanized three days after treatment, and to analyze sonoselective transfection efficacy, mice were euthanized two days after treatment. Prior to euthanasia, mice were perfused with ice‐cold 1x PBS, and tumor bearing brain quadrants were processed into single‐cell suspensions using the Adult Brain Dissociation Kit according as directed by the manufacturer (Miltenyi Biotec, Catalog #: 130‐107‐677). Cells were then stained for viability using the Live/Dead Fixable Blue dye (1:1000, Thermo Fisher Scientific, Catalog #: L23105). After washing, cells were then incubated with FcBlock, washed, and stained with aCD31 (1:100, Thermo Fisher Scientific, Catalog #: RM5228, RRID: AB_10373114), aCD45 (1:100, Thermo Fisher Scientific, Catalog #: A15395, RRID: AB_2534409), aEphA2 (1:200, Thermo Fisher Scientific, Catalog #: MA5‐40937, RRID: AB_2898698), ASCA‐2 (Miltenyi Biotec, Catalog #: 130‐116‐243, RRID: AB_2727421) aCD146 (1:50, Thermo Fisher Scientific, Catalog #: 25‐1469‐42, RRID: AB_2848308), and aH‐2Kk (1:10, Miltenyi Biotec, Catalog #: 130‐102‐346, RRID: AB_2659836). Cells were washed again and resuspended for analysis. Data were acquired using the 5‐laser Aurora Borealis spectral cytometer, and analysis was performed using the FCS Express software.

### 4.14 Immunofluorescent Imaging

To evaluate H‐2Kk expression, two days after delivery of SH‐NPs loaded with either a U‐H‐2Kk plasmid (Miltenyi Biotec, Plasmid #: 130‐092‐083) or CD144‐H‐2Kk plasmid (VectorBuilder, Plasmid #: VB240821‐1392jvd), mice were anesthetized and perfused with ice cold PBS and 4% paraformaldehyde. Brain samples were embedded in OCT (Thermo Fisher Scientific, Catalog #: 23‐730‐571) and frozen on dry ice. Frozen blocks were stored at ‐80°C before sectioning and staining. Cryo‐sections were cut at 20 um thick. The mounted sections were incubated with blocking solution [(1% NGS (Sigma‐Aldrich, Catalog #:578 NS02L) in 2% BSA (Jackson ImmunoResearch, Catalog #: 001‐000‐162) and 0.1% Tween 20579 (Thermo Fisher Scientific, Catalog #: BP337‐100) in PBS] for 1 h at room temperature. Sections were incubated overnight at 4°C with Alexa Fluor 594 anti‐mouse CD31 (1:200, BioLegend, Catalog #: 102520, RRID: AB_2563319) in antibody solution (2% BSA and 0.1% Tween 20 in PBS).

For profiling of resident memory T cells, on day 62 post‐inoculation, the mouse presenting with complete tumor eradication was anesthetized and perfused with ice‐cold PBS followed by 10% zinc fixative. Brain samples were embedded in OCT and frozen on dry ice. Frozen blocks were stored at ‐80°C before sectioning and staining. Cryo‐sections were cut with a 20 µm thickness. Sections were incubated overnight at 4°C with rabbit anti‐CD69 (1:500, Abcam, Catalog #: ab322534), Alexa Fluor 594 anti‐mouse CD103 (1:50, BioLegend, Catalog #: 121428, RRID: AB_2565571), and APC/Fire 750 anti‐mouse CD45 (1:800, BioLegend, Catalog #: 103154, RRID: AB_2572116) in antibody solution (2% BSA and 0.1% Tween 20 in PBS). After washing 3x for 10 min in 0.1% Tween 20 PBS, the sections were incubated with donkey anti‐rabbit‐Alexa Fluor 488 (1:800, Thermo Fisher Scientific, Catalog #: A‐21206, RRID: AB_2535792).

For either analysis, after washing 3x for 10 min in 0.1% Tween 20 PBS, sections were incubated for 20 min at room temp with DAPI (1:1000, Thermo Fisher Scientific, Catalog #: 62248). After final washes in PBS, sections were sealed with ProLong Gold antifade reagent (Thermo Fisher Scientific, Catalog #: P36930) and cover slipped with cover glass for confocal imaging using the Stellaris5 confocal microscope (Leica Microsystems, RRID: SCR_024671) and a 64x objective. Co‐localization of H‐2Kk and CD31 expression was assessed manually by using ImageJ (RRID:SCR_003070). Briefly, fluorescence channels were separated and independently thresholded to remove background signal. Overlapping H‐2Kk+ and CD31+ signal was identified using the Image Calculator “AND” function. The area of overlap was calculated using the “Measure” function, and its value was divided by the value of total H‐2Kk+ area to determine the fraction of H‐2kk expression within endothelial cells.

### 4.15 Quantification of Off‐Target Expression of E2‐Crimson

Two days after delivery of SH‐NPs encapsulating an E2‐Crimson plasmid (Takara Bio, Catalog #: 631981), mice were euthanized and perfused with ice‐cold PBS. Major organs including brain, heart, lungs, liver, spleen, and kidneys were harvested, protected from light, and stored in an ice‐cold 1x PBS solution. Organs were arranged on black paper and imaged using the LagoX optical imaging system.

### 4.16 Quantification of In Vivo Chemokine Expression

Fourteen days after inoculation, mice were separated into two groups and treated accordingly: CMV‐GFP: SH‐NPs encapsulating CMV‐GFP reporter plasmid delivered via FUS, CMV‐GFP‐CD144‐CXCL9: FUS‐mediated delivery of SH‐NPs encapsulating CD144‐CXCL9 plasmid with CMV‐GFP reporter. Two days later, brains were harvested and processed for flow cytometry using the Adult Brain Dissociation Kit. Cells were stained with Live/Dead Fixable Blue dye (1:1000, Thermo Fisher Scientific, Catalog #: L23105), washed, incubated with FcBlock, and washed again. Surface markers were stained using aCD45 (1:100, Thermo Fisher Scientific, Catalog #: A15395, RRID: AB_2534409), aCD31 (1:100, Thermo Fisher Scientific, Catalog #: RM5228, RRID: AB_10373114), and aEphA2 (1:200, Thermo Fisher Scientific, Catalog #: MA5‐40937, RRID: AB_2898698). After washing, cells were fixed and permeabilized for 10 min using ice‐cold 90% methanol. After washing again, cells were stained with aCXCL9 (1:200, Biolegend, Catalog #: 515606, RRID: AB_1877135), washed, and resuspended for analysis. Data were acquired using the 5‐laser Aurora Borealis spectral cytometer, and analysis was performed using the FCS Express software.

### 4.17 Sonoselective Chemokine Transfection

Fourteen days after inoculation, mice were separated into three groups and treated accordingly: FUS alone: FUS treatment of tumors, CMV‐GFP: SH‐NPs encapsulating CMV‐GFP reporter plasmid delivered via FUS, CMV‐GFP‐CD144‐CXCL9: FUS‐mediated delivery of SH‐NPs encapsulating CD144‐CXCL9 plasmid with CMV‐GFP reporter. Three days after FUS treatment, tumor‐bearing brain quadrants were harvested and placed in ice‐cold RPMI+FBS. The tissue was minced using a sterile surgical blade and passed through an 18‐gauge needle for homogenization. Samples were then enzymatically digested with Collagenase A (1 mg/mL; Sigma‐Aldrich, Catalog#: 10103578001) and DNase I (1 mg/mL; Sigma‐Aldrich, Catalog #: 10104159001) at 37°C for 20 min and then passed through a 70 µm strainer (Corning, Catalog #: 431751) and washed with RPMI+FBS. A Percoll (Sigma‐Aldrich, Catalog #: P1644) gradient was used to remove myelin by resuspending the cell pellet in 10 mL of 40% Percoll and centrifuging for 10 min at 650 × g. The remaining cell pellet was resuspended in 10 mL RPMI+FBS and was further centrifuged at 1800 RPM for 10 min. Cells were then stained with Live/Dead Fixable Blue dye (1:1000, Thermo Fisher Scientific, Catalog #: L23105), washed, incubated with FcBlock, and washed again. Surface markers were stained using aCD45 (1:100, ThermoFisher, Catalog #: A15395, RRID: AB_2534409), aCD4 (1:400, Thermo Fisher Scientific, Catalog #: M001T02B06‐A, RRID: AB_3098875), aCD8 (1:200, Thermo Fisher Scientific, Catalog #: 48‐0081‐82, RRID: AB_1272198), aCD3 (1:100, BD Biosciences, Catalog #: 741319, RRID: AB_2870837), aNK1.1 (1:200, BioLegend, Catalog #: 108745, RRID: AB_2563286). After washing, cells were fixed and permeabilized using the Foxp3/Transcription Factor Staining Buffer Set (Thermo Fisher Scientific, Catalog #: 00‐5523‐00) overnight at 4°C, according to the manufacturer. Cells were then washed and stained using aFOXP3 (1:200, Thermo Fisher Scientific, Catalog #: 606‐5773‐82, RRID: AB_2896288). Cells were washed once more and resuspended in 200 µL FACS buffer (0.2% BSA and 2 mM EDTA in 1X PBS) and 25 uL of CountBright Absolute Counting Beads (Thermo Fisher Scientific, Catalog #: C36950). Data were acquired using the 5‐laser Aurora Borealis spectral cytometer, and analysis was performed using the FCS Express software.

### 4.18 αPD‐1 Treatment of Tumor‐Bearing Mice

CXCL9 plasmid‐bearing SH‐NPs were delivered to GL261‐Luc2 tumor‐bearing mice at the time of FUS treatment as described above. Mice were treated with i.p. injections of 200ug of either IgG control (Ichorbio, Catalog #: ICH2244, RRID: AB_2921379) or αPD‐1 (BioXCell, Catalog #: BE0146, RRID: AB_10949053) antibodies on days 15, 17, 19, and 21 post‐tumor inoculation. Mice were weighed every day starting on day 14 and monitored until euthanasia. Mice were weighed every day starting on day 14 and monitored until euthanasia. Mouse weight changes are provided in Figure S9.

### 4.19 Statistical Analysis

All data are reported as mean ± standard error of the mean (SEM). The “n” values per group are made evident by individual data points or by text in figure captions, with all “n” values referring to the number of mice. Statistical significance was assessed at *p* < 0.05 for all experiments, and details of statistical testing are explained in the figure legends (GraphPad Prism 10).

## Author Contributions

ACD and RJP conceptualized the study. ACD conducted the experiments with the aid of CMG, VRB, KMN, JT, KP, and JS. ACD, RJP, and VRB designed the figures and wrote the manuscript. ACD, CMG, VRB, KMN, JT, KP, JS, NDS, and RJP edited the manuscript. CMG, NDS, and RJP provided funding support. All authors approved the manuscript.

## Conflicts of Interest

The authors declare no conflicts of interest.

## Supporting information




**Supporting File**: advs76997‐sup‐0001‐SuppMat.pdf.

## Data Availability

The data that support the findings of this study are available from the corresponding author upon reasonable request.

## References

[1] C. Fernandes , A. Costa , and L. Osório , Glioblastoma (Exon Publication, 2017), 197, 10.15586/codon.glioblastoma.2017.

[2] L. Rong , N. Li , and Z. Zhang , “Emerging Therapies for Glioblastoma: Current State and Future Directions,” Journal of Experimental & Clinical Cancer Research 41, no. 1 (2022): 142, 10.1186/s13046-022-02349-7. PMC9013078 35428347

[3] A. Omuro and L. M. DeAngelis , “Glioblastoma and Other Malignant Gliomas,” JAMA 310, no. 17 (2013): 1842, 10.1001/jama.2013.280319. 24193082

[4] T. S. Van Solinge , L. Nieland , E. A. Chiocca , and M. L. D. Broekman , “Advances in Local Therapy for Glioblastoma—Taking the Fight to the Tumour,” Nature Reviews Neurology 18 (2022): 221–236. 10.1038/s41582-022-00621-0 PMC10359969 35277681

[5] C. Pilotto Heming , W. Muriithi , L. Wanjiku Macharia , P. Niemeyer Filho , V. Moura‐Neto , and V. Aran , “P‐glycoprotein and Cancer: What Do We Currently Know?,” Heliyon 8 (2022): 11171. 10.1016/j.heliyon.2022.e11171 PMC9618987 36325145

[6] D. Wu , Q. Chen , X. Chen , F. Han , Z. Chen , and Y. Wang , “The Blood–Brain Barrier: Structure, Regulation and Drug Delivery,” Signal Transduction and Targeted Therapy 8 (2023): 217. 10.1038/s41392-023-01481-w PMC10212980 37231000

[7] J. Wu , “The Enhanced Permeability and Retention (Epr) Effect: The Significance of the Concept and Methods to Enhance Its Application,” Journal of Personalized Medicine 11 (2021): 771. 10.3390/jpm11080771 PMC8402171 34442415

[8] U. Prabhakar , H. Maeda , and R. K. Jain , “Challenges and Key Considerations of the Enhanced Permeability and Retention Effect for Nanomedicine Drug Delivery in Oncology,” Cancer Research 73, no. 8 (2013): 2412–2417, 10.1158/0008-5472.CAN-12-4561. PMC3916009 23423979

[9] R. Callaghan , F. Luk , and M. Bebawy , “Inhibition of the Multidrug Resistance P‐Glycoprotein: Time for a Change of Strategy?,” Drug Metabolism and Disposition 42, no. 4 (2014): 623–631, 10.1124/dmd.113.056176. PMC3965902 24492893

[10] D. Waghray and Q. Zhang , “Inhibit or Evade Multidrug Resistance P‐Glycoprotein in Cancer Treatment,” Journal of Medicinal Chemistry 61, no. 12 (2018): 5108–5121, 10.1021/acs.jmedchem.7b01457. PMC6281405 29251920

[11] H. Lin , C. Liu , A. Hu , D. Zhang , H. Yang , and Y. Mao , “Understanding the Immunosuppressive Microenvironment of Glioma: Mechanistic Insights and Clinical Perspectives,” Journal of Hematology & Oncology 17 (2024): 31. 10.1186/s13045-024-01544-7 PMC11077829 38720342

[12] R. Luciano , G. Battafarano , and R. Saracino , “New Perspectives in Glioblastoma: Nanoparticles‐based Approaches,” Current Cancer Drug Targets 17, no. 3 (2016): 203–220, 10.2174/1568009616666160813190732. 27528362

[13] P. Játiva and V. Ceña , “Use of Nanoparticles for Glioblastoma Treatment: A New Approach,” Nanomedicine 12 (2017): 2533–2554. 10.2217/nnm-2017-0223 28952878

[14] F. Alexis , E. Pridgen , L. K. Molnar , and O. C. Farokhzad , “Factors Affecting the Clearance and Biodistribution of Polymeric Nanoparticles,” Molecular Pharmaceutics 5, no. 4 (2008): 505–515, 10.1021/mp800051m. PMC2663893 18672949

[15] X. Wang , D. Niu , C. Hu , and P. Li , “Polyethyleneimine‐Based Nanocarriers for Gene Delivery,” Current Pharmaceutical Design 21, no. 42 (2015): 6140–6156, 10.2174/1381612821666151027152907. 26503146

[16] N. Sayed , P. Allawadhi , and A. Khurana , “Gene Therapy: Comprehensive Overview and Therapeutic Applications,” Life Sciences 294 (2022): 120375. 10.1016/j.lfs.2022.120375 35123997

[17] H. Zu and D. Gao , “Non‐Viral Vectors in Gene Therapy: Recent Development, Challenges, and Prospects,” The AAPS Journal 23 (2021): 78. 10.1208/s12248-021-00608-7 PMC8171234 34076797

[18] T. J. Thomas , H. A. Tajmir‐Riahi , and C. K. S. Pillai , “Biodegradable Polymers for Gene Delivery,” Molecules 24, no. 20 (2019): 3744, 10.3390/molecules24203744. PMC6832905 31627389

[19] R. Kumar , C. F. Santa Chalarca , and M. R. Bockman , “Polymeric Delivery of Therapeutic Nucleic Acids,” Chemical Reviews 121 (2021): 11527–11652. 10.1021/acs.chemrev.0c00997 33939409

[20] M. A. Subhan , S. S. K. Yalamarty , N. Filipczak , F. Parveen , and V. P. Torchilin , “Recent Advances in Tumor Targeting via EPR Effect for Cancer Treatment,” Journal of Personalized Medicine 11 (2021): 571. 10.3390/jpm11060571 PMC8234032 34207137

[21] R. S. D'Amico , M. K. Aghi , M. A. Vogelbaum , and J. N. Bruce , “Convection‐enhanced Drug Delivery for Glioblastoma: A Review,” Journal of Neuro‐Oncology 151 (2021): 415–427. 10.1007/s11060-020-03408-9 PMC8034832 33611708

[22] T. H. Ung , H. Malone , P. Canoll , and J. N. Bruce , “Convection‐enhanced Delivery for Glioblastoma: Targeted Delivery of Antitumor Therapeutics,” CNS Oncology 4 (2015): 225–234. 10.2217/cns.15.12 PMC6088338 26103989

[23] K. F. Timbie , B. P. Mead , and R. J. Price , “Drug and Gene Delivery Across the Blood–brain Barrier with Focused Ultrasound,” Journal of Controlled Release 219 (2015): 61–75. 10.1016/j.jconrel.2015.08.059 PMC4656107 26362698

[24] C. M. Gorick , V. R. Breza , and K. M. Nowak , “Applications of Focused Ultrasound‐mediated Blood‐brain Barrier Opening,” Advanced Drug Delivery Reviews 191 (2022): 114583. 10.1016/j.addr.2022.114583 PMC9712235 36272635

[25] C. Bérard , C. Truillet , and B. Larrat , “Anticancer Drug Delivery by Focused Ultrasound‐mediated Blood‐brain/Tumor Barrier Disruption for Glioma Therapy: from Benchside to Bedside,” Pharmacology & Therapeutics 250 (2023): 108518. 10.1016/j.pharmthera.2023.108518 37619931

[26] B. P. Mead , P. Mastorakos , J. S. Suk , A. L. Klibanov , J. Hanes , and R. J. Price , “Targeted Gene Transfer to the Brain via the Delivery of Brain‐penetrating DNA Nanoparticles with Focused Ultrasound,” Journal of Controlled Release 223 (2016): 109–117, 10.1016/j.jconrel.2015.12.034. PMC4739627 26732553

[27] C. T. Curley , B. P. Mead , and K. Negron , “Augmentation of Brain Tumor Interstitial Flow via Focused Ultrasound Promotes Brain‐penetrating Nanoparticle Dispersion and Transfection,” Science Advances 6 (2020): aay1344. 10.1126/sciadv.aay1344 PMC7195188 32494662

[28] K. F. K. F. Timbie , U. Afzal , and A. Date , “MR Image‐guided Delivery of Cisplatin‐loaded Brain‐penetrating Nanoparticles to Invasive Glioma with Focused Ultrasound,” Journal of Controlled Release 263 (2017): 120–131, 10.1016/j.jconrel.2017.03.017. PMC5593770 28288892

[29] E. Nance , K. Timbie , and G. W. Miller , “Non‐invasive Delivery of Stealth, Brain‐penetrating Nanoparticles across the Blood − Brain Barrier Using MRI‐guided Focused Ultrasound,” Journal of Controlled Release 189 (2014): 123–132, 10.1016/j.jconrel.2014.06.031. PMC4125545 24979210

[30] V. M. Lu and T. N. Niazi , “Clinical Trials of Focused Ultrasound for Brain Tumors,” Cancers 17 (2025): 513. 10.3390/cancers17030513 PMC11817304 39941880

[31] K. Banerjee , F. J. Núñez , and S. Haase , “Current Approaches for Glioma Gene Therapy and Virotherapy,” Frontiers in Molecular Neuroscience 14 (2021): 621831, 10.3389/fnmol.2021.621831. PMC8006286 33790740

[32] T. Kang , M. Jiang , and D. Jiang , “Enhancing Glioblastoma‐Specific Penetration by Functionalization of Nanoparticles with an Iron‐Mimic Peptide Targeting Transferrin/Transferrin Receptor Complex,” Molecular Pharmaceutics 12, no. 8 (2015): 2947–2961, 10.1021/acs.molpharmaceut.5b00222. 26149889

[33] T. Kang , Q. Zhu , and D. Jiang , “Synergistic Targeting Tenascin C and Neuropilin‐1 for Specific Penetration of Nanoparticles for Anti‐glioblastoma Treatment,” Biomaterials 101 (2016): 60–75, 10.1016/j.biomaterials.2016.05.037. 27267628

[34] L. Zhao , H. Chen , and L. Lu , “Design and Screening of a Novel Neuropilin‐1 Targeted Penetrating Peptide for Anti‐angiogenic Therapy in Glioma,” Life Sciences 270 (2021): 119113, 10.1016/j.lfs.2021.119113. 33508290

[35] C. Zhan , Q. Meng , Q. Li , L. Feng , J. Zhu , and W. Lu , “Cyclic RGD–Polyethylene Glycol–Polyethylenimine for Intracranial Glioblastoma‐Targeted Gene Delivery,” Chemistry—An Asian Journal 7, no. 1 (2012): 91–96, 10.1002/asia.201100570. 22072592

[36] L. Lin , J. Chen , and Z. Guo , “Exploring the in Vivo Fates of RGD and PEG Modified PEI/DNA Nanoparticles by Optical Imaging and Optoacoustic Imaging,” RSC Advances 6 (2016): 112552–112561.

[37] L. Peng , Y. Liang , and X. Zhong , “<p>Aptamer‐Conjugated Gold Nanoparticles Targeting Epidermal Growth Factor Receptor Variant III for the Treatment of Glioblastoma</p>,” International Journal of Nanomedicine 15 (2020): 1363–1372, 10.2147/IJN.S238206. PMC7053811 32184591

[38] A. G. Torres and M. J. Gait , “Exploiting Cell Surface Thiols to Enhance Cellular Uptake,” Trends in Biotechnology 30, no. 4 (2012): 185–190, 10.1016/j.tibtech.2011.12.002. 22260747

[39] A. Rodríguez‐Camacho , J. G. Flores‐Vázquez , and J. Moscardini‐Martelli , “Glioblastoma Treatment: State‐of‐the‐Art and Future Perspectives,” International Journal of Molecular Sciences 23 (2022): 7207. 10.3390/ijms23137207 PMC9267036 35806212

[40] N. Hock , G. F. Racaniello , S. Aspinall , N. Denora , V. V. Khutoryanskiy , and A. Bernkop‐Schnürch , Thiolated Nanoparticles for Biomedical Applications: Mimicking the Workhorses of Our Body (John Wiley and Sons Inc, 2022). 10.1002/advs.202102451 PMC8728822 34773391

[41] Y. Lee , H. Mo , and H. Koo , “Visualization of the Degradation of a Disulfide Polymer, Linear Poly(ethylenimine sulfide), for Gene Delivery,” Bioconjugate Chemistry 18, no. 1 (2007): 13–18, 10.1021/bc060113t. 17226953

[42] Q. Peng , C. Hu , J. Cheng , Z. Zhong , and R. Zhuo , “Influence of Disulfide Density and Molecular Weight on Disulfide Cross‐Linked Polyethylenimine as Gene Vectors,” Bioconjugate Chemistry 20, no. 2 (2009): 340–346, 10.1021/bc800451j. 19191568

[43] Q. Peng , Z. Zhong , and R. Zhuo , “Disulfide Cross‐Linked Polyethylenimines (PEI) Prepared via Thiolation of Low Molecular Weight PEI as Highly Efficient Gene Vectors,” Bioconjugate Chemistry 19, no. 2 (2008): 499–506, 10.1021/bc7003236. 18205328

[44] M. Muthiah , H. L. Che , and S. Kalash , “Formulation of Glutathione Responsive Anti‐proliferative Nanoparticles from Thiolated Akt1 siRNA and Disulfide‐crosslinked PEI for Efficient Anti‐cancer Gene Therapy,” Colloids and Surfaces B: Biointerfaces 126 (2015): 322–327, 10.1016/j.colsurfb.2014.12.022. 25576812

[45] G. Q. Lin , W. J. Yi , Q. Liu , X. J. Yang , and Z. G. Zhao , “Aromatic Thioacetal‐bridged ROS‐responsive Nanoparticles as Novel Gene Delivery Vehicles,” Molecules 23 (2018): 2061. 10.3390/molecules23082061 PMC6225261 30126108

[46] X. Kong , Y. Sun , and Q. Zhang , “Specific Tumor Cell Detection by a Metabolically Targeted Aggregation‐induced Emission‐based Gold Nanoprobe,” ACS Omega 7 (2022): 18073–18084. 10.1021/acsomega.2c01494 PMC9161387 35664593

[47] G. T. Zugates , D. G. Anderson , S. R. Little , I. E. B. Lawhorn , and R. Langer , “Synthesis of Poly(β‐amino ester)s with Thiol‐Reactive Side Chains for DNA Delivery,” Journal of the American Chemical Society 128, no. 39 (2006): 12726–12734, 10.1021/ja061570n. 17002366

[48] S. M. Shiels , K. D. Solomon , M. Pilia , M. R. Appleford , and J. L. Ong , “BMP‐2 Tethered Hydroxyapatite for Bone Tissue Regeneration: Coating Chemistry and Osteoblast Attachment,” Journal of Biomedical Materials Research Part A 100A, no. 11 (2012): 3117–3123, 10.1002/jbm.a.34241. 22815074

[49] M. Jester , R. M. Haley , and M. M. Billingsley , “Ionizable Lipid Nanoparticles with Functionalized PEG‐lipids Increase Retention in the Tumor Microenvironment,” Molecular Therapy Methods & Clinical Development 33, no. 2 (2025): 101457, 10.1016/j.omtm.2025.101457. PMC12049828 40321415

[50] A. J. Slezak , K. Chang , and T. N. Beckman , “Cysteine‐binding Adjuvant Enhances Survival and Promotes Immune Function in a Murine Model of Acute Myeloid Leukemia,” Blood Advances 8, no. 7 (2024): 1747–1759, 10.1182/bloodadvances.2023012529. PMC10985806 38324726

[51] A. J. Slezak , A. Mansurov , and M. M. Raczy , “Tumor Cell‐Surface Binding of Immune Stimulating Polymeric Glyco‐Adjuvant via Cysteine‐Reactive Pyridyl Disulfide Promotes Antitumor Immunity,” ACS Central Science 8, no. 10 (2022): 1435–1446, 10.1021/acscentsci.2c00704. PMC9615125 36313164

[52] J. R. Kane , J. Miska , J. S. Young , D. Kanojia , J. W. Kim , and M. S. Lesniak , “Sui Generis: Gene Therapy and Delivery Systems for the Treatment of Glioblastoma,” Neuro‐Oncology 17, 2 (2015): ii24–ii36, 10.1093/neuonc/nou355. PMC4483038 25746089

[53] D. Khaitan , P. L. Reddy , and N. Ningaraj , “Targeting Brain Tumors with Nanomedicines: Overcoming Blood Brain Barrier Challenges,” Current Clinical Pharmacology 13, no. 2 (2018): 110–119, 10.2174/1574884713666180412150153. 29651960

[54] C. M. Gorick , A. S. Mathew , and W. J. Garrison , “Sonoselective Transfection of Cerebral Vasculature without Blood–brain Barrier Disruption,” Proceedings of the National Academy of Sciences 117, no. 11 (2020): 5644–5654, 10.1073/pnas.1914595117. PMC7084076 32123081

[55] M. Alberto , A. C. Paiva‐Santos , F. Veiga , and P. C. Pires , “Lipid and Polymeric Nanoparticles: Successful Strategies for Nose‐to‐Brain Drug Delivery in the Treatment of Depression and Anxiety Disorders,” Pharmaceutics 14, no. 12 (2022): 2742, 10.3390/pharmaceutics14122742. PMC9783528 36559236

[56] O. V. Chumakova , A. V. Liopo , and V. G. Andreev , “Composition of PLGA and PEI/DNA Nanoparticles Improves Ultrasound‐mediated Gene Delivery in Solid Tumors in Vivo,” Cancer Letters 261, no. 2 (2008): 215–225, 10.1016/j.canlet.2007.11.023. 18164806

[57] C. Gallops , J. Ziebarth , and Y. Wang , “A Polymer Physics Perspective on Why PEI Is an Effective Nonviral Gene Delivery Vector." Polymers in Therapeutic Delivery,” American Chemical Society 1350 (2020): 1–10.

[58] E. Nance , C. Zhang , T. Y. Shih , Q. Xu , B. S. Schuster , and J. Hanes , “Brain‐Penetrating Nanoparticles Improve Paclitaxel Efficacy in Malignant Glioma Following Local Administration,” ACS Nano 8, no. 10 (2014): 10655–10664, 10.1021/nn504210g. PMC4212792 25259648

[59] B. E. O'Neill , H. Vo , M. Angstadt , K. P. C. Li , T. Quinn , and V. Frenkel , “Pulsed High Intensity Focused Ultrasound Mediated Nanoparticle Delivery: Mechanisms and Efficacy in Murine Muscle,” Ultrasound in Medicine & Biology 35 (2009): 416–424. 10.1016/j.ultrasmedbio.2008.09.021 PMC2668521 19081668

[60] S. Chen , A. Nazeri , and H. Baek , “A Review of Bioeffects Induced by Focused Ultrasound Combined with Microbubbles on the Neurovascular Unit,” Journal of Cerebral Blood Flow & Metabolism 42, no. 1 (2021): 3–26, 10.1177/0271678X211046129. PMC8721781 34551608

[61] P. G. Durham , A. Butnariu , R. Alghorazi , G. Pinton , V. Krishna , and P. A. Dayton , “Current Clinical Investigations of Focused Ultrasound Blood‐brain Barrier Disruption: a Review,” Neurotherapeutics 21, no. 3 (2024): e00352, 10.1016/j.neurot.2024.e00352. PMC11044032 38636309

[62] P. Mastorakos , C. Zhang , and S. Berry , “Highly PEGylated DNA Nanoparticles Provide Uniform and Widespread Gene Transfer in the Brain,” Advanced Healthcare Materials 4, no. 7 (2015): 1023–1033, 10.1002/adhm.201400800. PMC4433405 25761435

[63] K. Negron , N. Khalasawi , and B. Lu , “Widespread Gene Transfer to Malignant Gliomas with In Vitro‐to‐In Vivo Correlation,” Journal of Controlled Release 303 (2019): 1–11, 10.1016/j.jconrel.2019.04.010. PMC6579670 30978431

[64] Y. Rui and J. J. Green , “Overcoming Delivery Barriers in Immunotherapy for Glioblastoma,” Drug Delivery and Translational Research 11, no. 6 (2021): 2302–2316, 10.1007/s13346-021-01008-2. PMC8164566 34053034

[65] M. Wagner , A. Krieger , and M. Minameyer , “Multiresponsive Polymer Nanoparticles Based on Disulfide Bonds,” Macromolecules 54, no. 6 (2021): 2899–2911, 10.1021/acs.macromol.1c00299.

[66] P. Wei , E. J. Cornel , and J. Du , “Ultrasound‐responsive Polymer‐based Drug Delivery Systems,” Drug Delivery and Translational Research 11, no. 4 (2021): 1323–1339, 10.1007/s13346-021-00963-0. PMC7989687 33761101

[67] Y. Zhang , Y. Li , S. Li , H. Zhang , and H. Ma , “In Situ Monitoring of the Effect of Ultrasound on the Sulfhydryl Groups and Disulfide Bonds of Wheat Gluten,” Molecules 23, no. 6 (2018): 1376, 10.3390/molecules23061376. PMC6100594 29875337

[68] X. Chen , D. A. Wolfe , and D. S. Bindu , “Functional Gene Delivery to and Across Brain Vasculature of Systemic AAVs With Endothelial‐Specific Tropism in Rodents and Broad Tropism in Primates,” Nature Communications 14 (2023): 3345. 10.1038/s41467-023-38582-7 PMC10250345 37291094

[69] T. Krolak , K. Y. Chan , and L. Kaplan , “A High‐efficiency AAV for Endothelial Cell Transduction Throughout the Central Nervous System,” Nature Cardiovascular Research 1, no. 4 (2022): 389–400, 10.1038/s44161-022-00046-4. PMC9103166 35571675

[70] T. M. H. Huynh , N. T. Tran , and Y. S. Lee , “Dysfunctional T Cell Reprogramming via Cascade‐Responsive Catalytic Nanosponges‐Mediated Tumor‐Specific Antigen Capture for Remote Glioblastoma Immunotherapy,” Advanced Functional Materials 36, no. 24 (2025): 23478, 10.1002/adfm.202523478.

[71] F. De Sanctis , S. Ugel , J. Facciponte , and A. Facciabene , The Dark Side of Tumor‐associated Endothelial Cells (Academic Press, 2018). 10.1016/j.smim.2018.02.002 29490888

[72] D. Klein , “The Tumor Vascular Endothelium as Decision Maker in Cancer Therapy,” Frontiers in Oncology 8 (2018): 367, 10.3389/fonc.2018.00367. PMC6139307 30250827

[73] Z. R. Huinen , E. J. M. Huijbers , J. R. van Beijnum , P. Nowak‐Sliwinska , and A. W. Griffioen , “Anti‐angiogenic Agents — Overcoming Tumour Endothelial Cell Anergy and Improving Immunotherapy Outcomes,” Nature Reviews Clinical Oncology 18, no. 8 (2021): 527–540, 10.1038/s41571-021-00496-y. 33833434

[74] A. N. Woods , A. L. Wilson , and N. Srivinisan , “Differential Expression of Homing Receptor Ligands on Tumor‐Associated Vasculature That Control CD8 Effector T‐cell Entry,” Cancer Immunology Research 5, no. 12 (2017): 1062–1073, 10.1158/2326-6066.CIR-17-0190. PMC6069521 29097419

[75] S. Y. Neo and A. Lundqvist , “The Multifaceted Roles of CXCL9 within the Tumor Microenvironment,” Advances in Experimental Medicine and Biology 1231 (2020): 45–51. 10.1007/978-3-030-36667-4_5 32060845

[76] C. A. von Roemeling , J. A. Patel , and S. L. Carpenter , “Adeno‐Associated Virus Delivered CXCL9 Sensitizes Glioblastoma to anti‐PD‐1 Immune Checkpoint Blockade,” Nature Communications 15 (2024): 58715. 10.1038/s41467-024-49989-1 PMC11245621 38997283

[77] S. Akter , R. Madhuvilakku , and A. K. Kar , “Reactive Oxygen Species (ROS) in Cancer: from Mechanism to Therapeutic Implications,” Signal Transduction and Targeted Therapy 11, no. 1 (2026): 111, 10.1038/s41392-026-02583-x. PMC13018652 41881983

