# Supplementary material for: Sonoselective Transfection of Glioma Endothelium
Source: Adv Sci (Weinh). 2026 Aug 5;13(60):e76997. doi: 10.1002/advs.76997 (PMC13440367; doi:10.1002/advs.76997)
Supplement: Supplementary file 1 [file ADVS-13-e76997-s001.pdf]

## *Supplemental Material For:*

# Sonoselective Transfection of Glioma Endothelium


**Anna C. Debski[1], Catherine M. Gorick[1], Victoria R. Breza[1], Jackson Tirrell[1], Krishnan Perumal[1], Katherine M. Nowak[1], Ji Song[1], Natasha D. Sheybani[1], and Richard J. Price[1,2]**

1. Dept. of Biomedical Engineering, University of Virginia, Charlottesville, VA
2. Radiology & Medical Imaging, University of Virginia, Charlottesville, VA

Corresponding Author:

Richard J. Price, Ph.D.
Department of Biomedical Engineering
Department of Radiology & Medical Imaging
Box 800759, Health System
University of Virginia
Charlottesville, VA 22908, USA
Telephone: (434) 924-0020
Email: rprice@virginia.edu
ORCID: 0000-0002-0237-2102

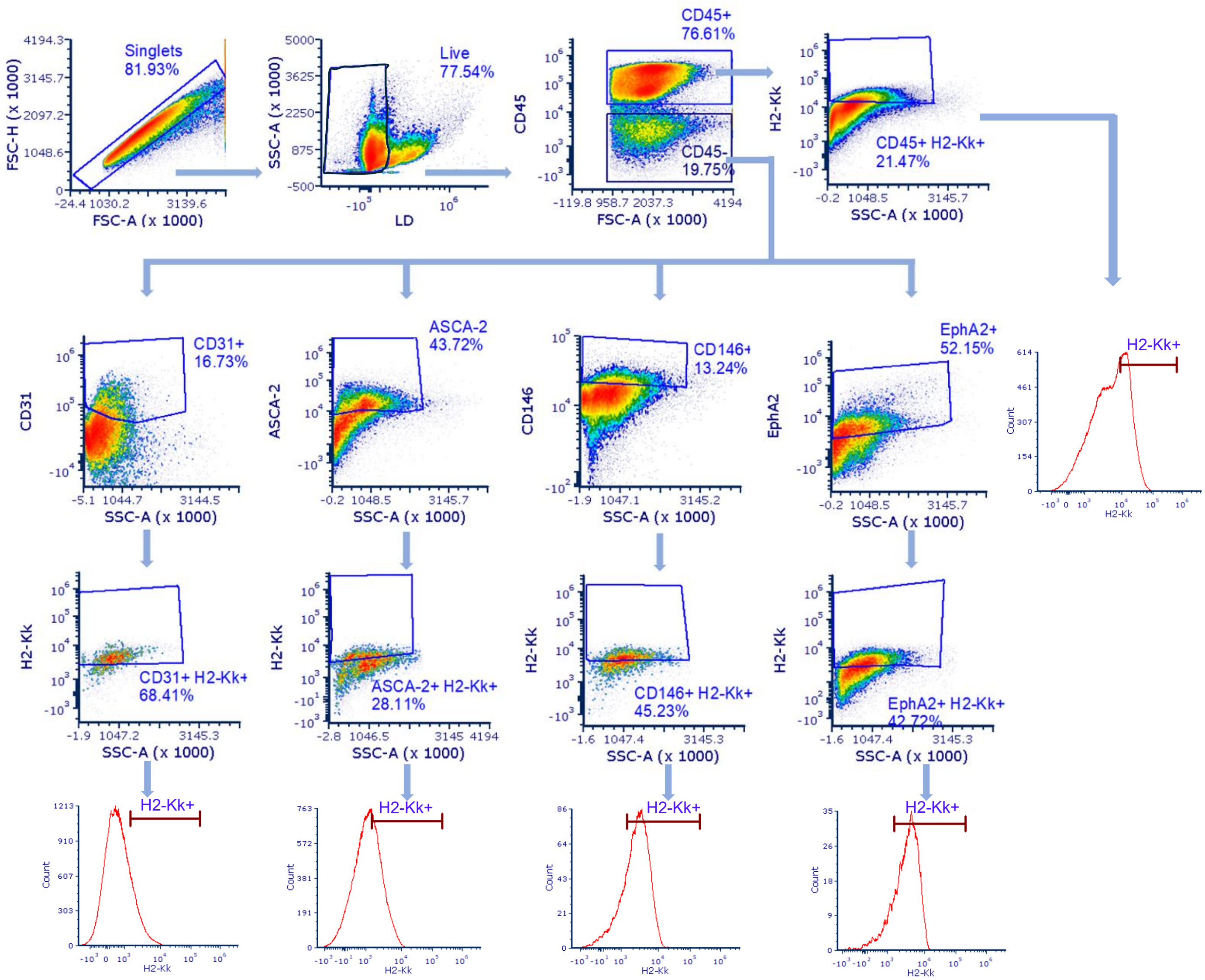


**Figure S1: Gating strategy for flow cytometry data following SH-NP delivery of reporter plasmids.**

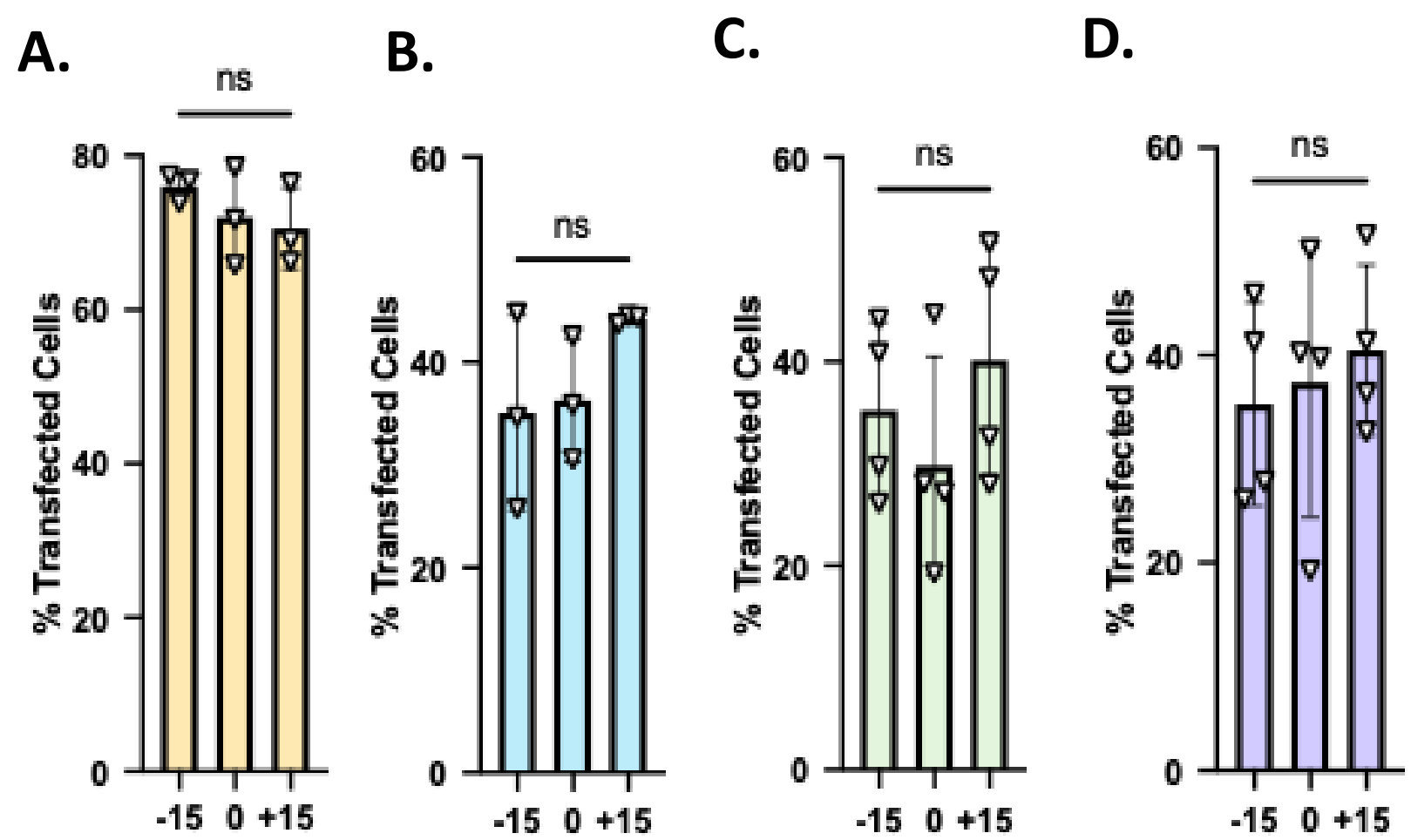


**Figure S2: Characterization of SH-NP transfection efficacy relative to FUS treatment time**. Influence timing of SH-NP delivery (15 min before (-15), at the time of (0) or 15 min after (-15)) relative to FUS treatment on percentage of transfected endothelial cells (A), astrocytes (B), pericytes (C) and leukocytes (D). +15: n=3, 0: n=3, -15: n=3; One-Way ANOVAs followed by Tukey's multiple comparison's tests.

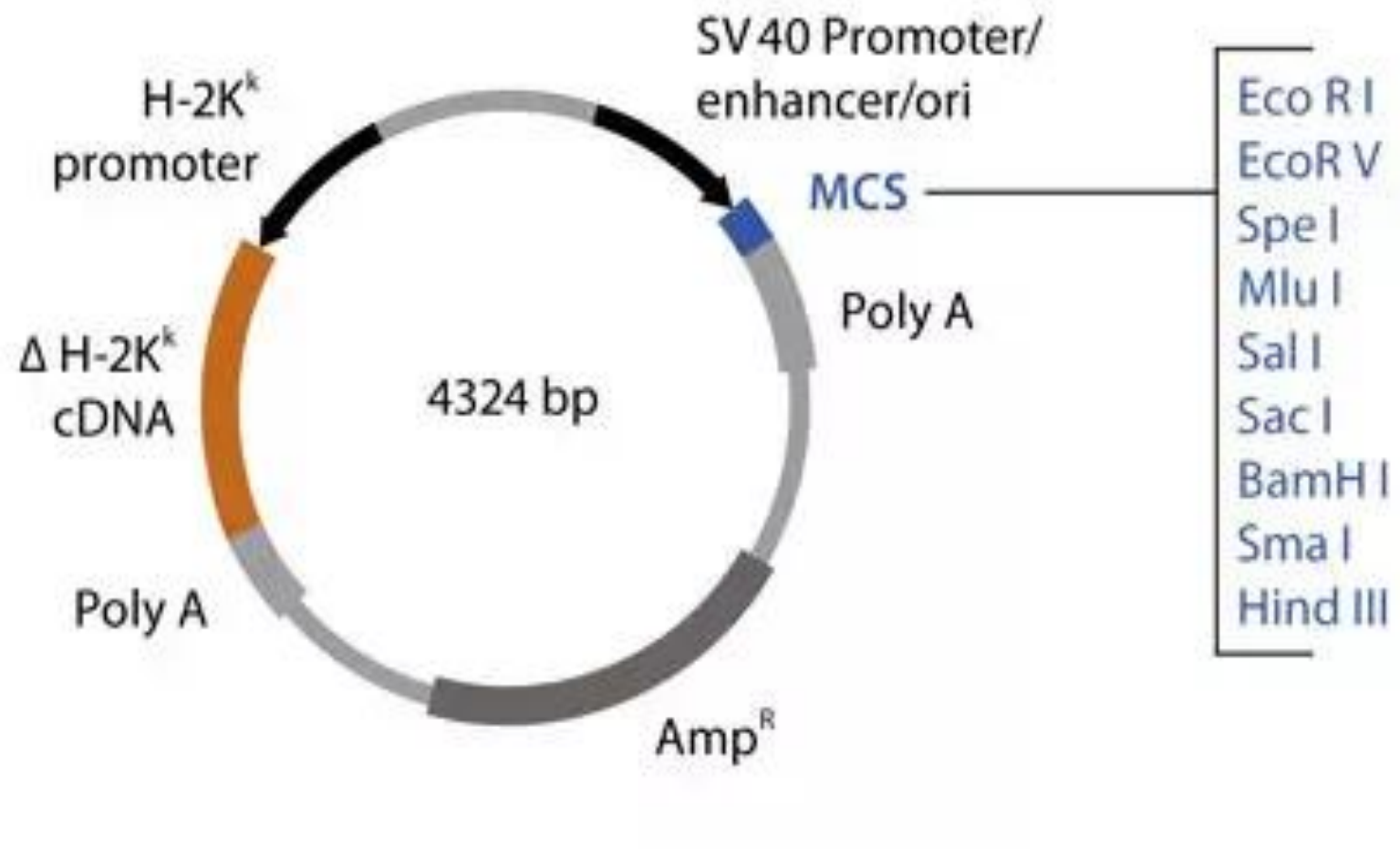


**Figure S3: Schematic of U-H-2Kk plasmid vector.**

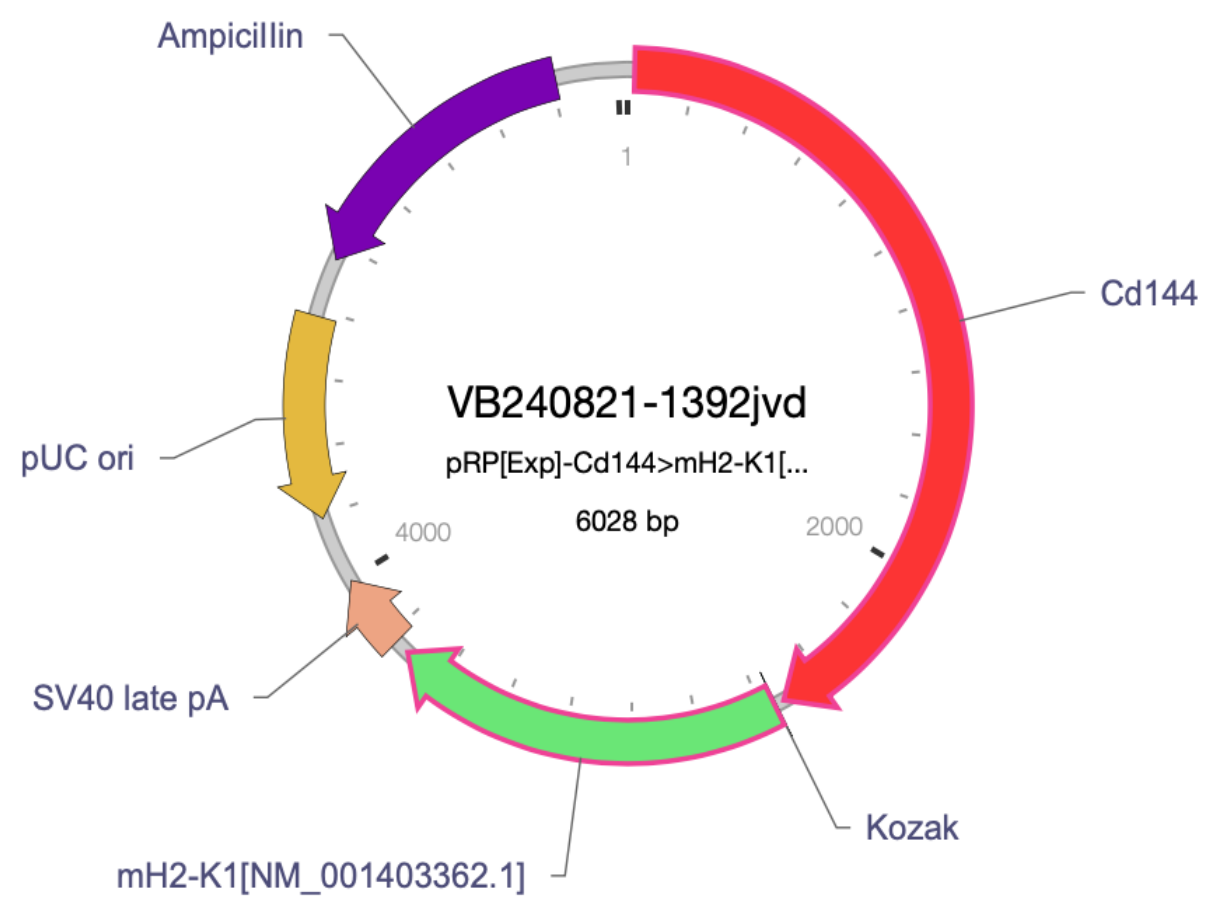


**Figure S4: Schematic of CD144-H-2Kk plasmid vector.**

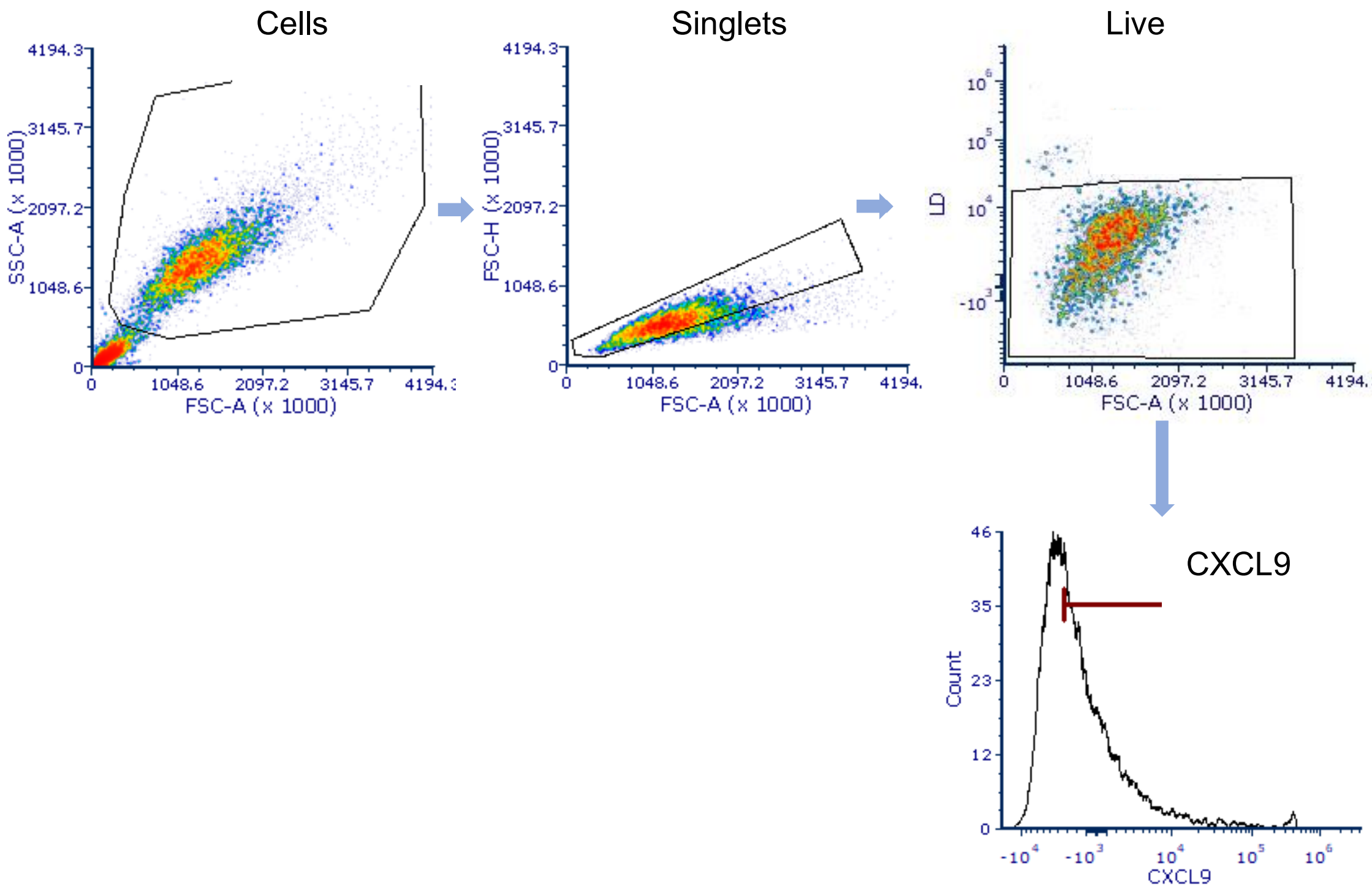


**Figure S5: Flow cytometry gating strategy used for quantification of CXCL9 expression in vitro.**

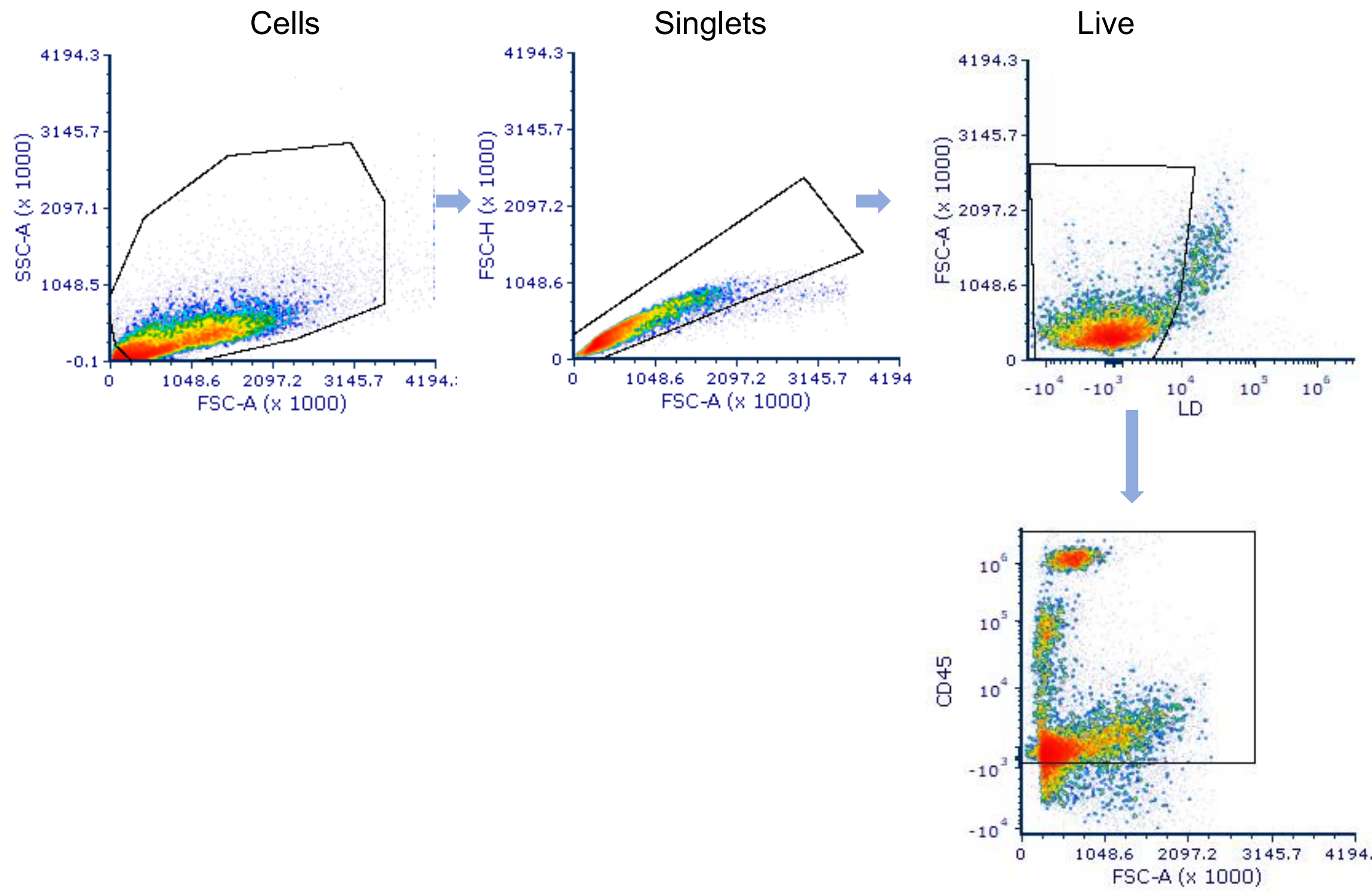


**Figure S6: Flow cytometry gating strategy used for quantifying percentage of T cells recruited to CXCL9-transfected bEnd.3 cells.**

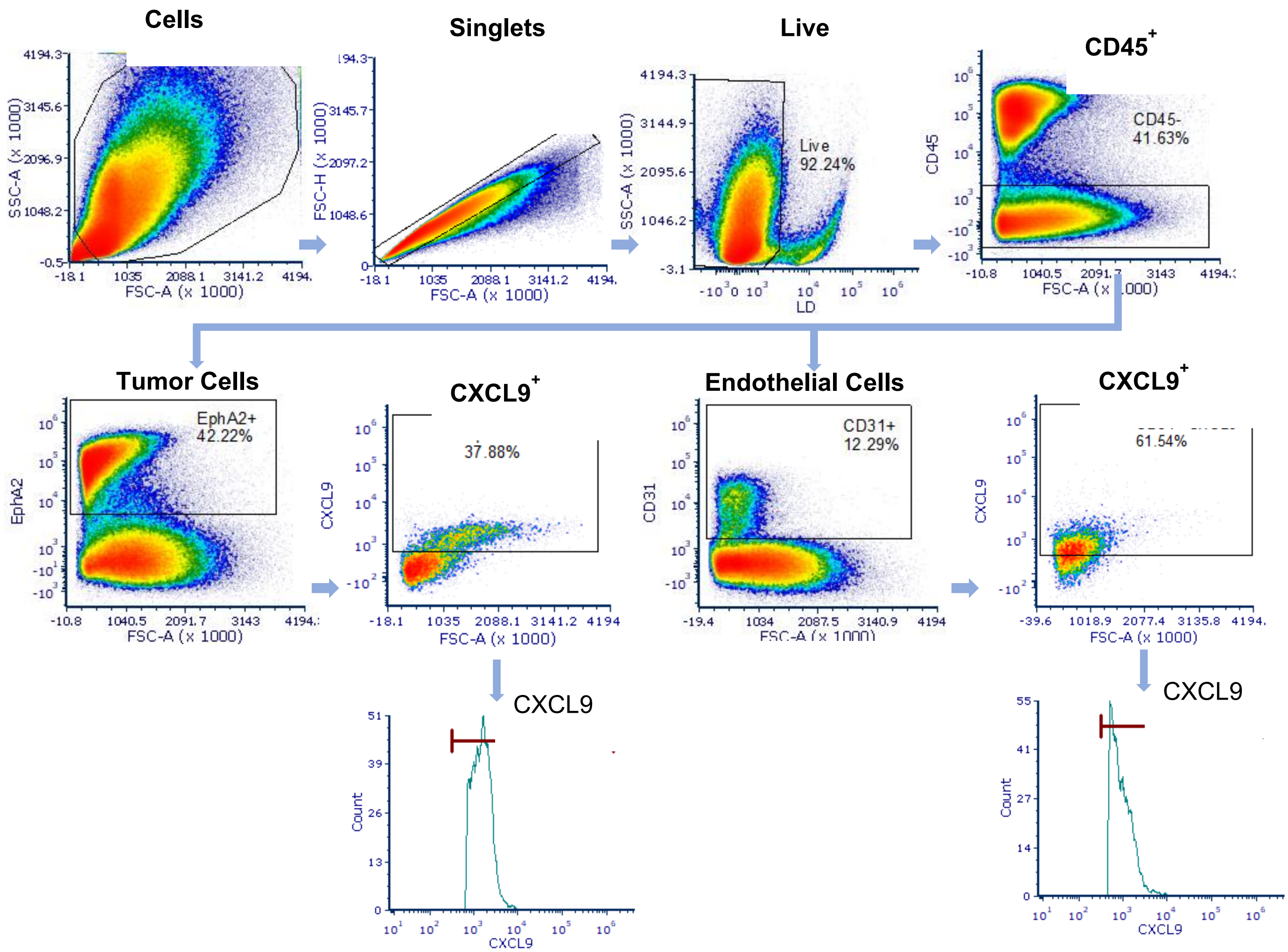


**Figure S7: Flow cytometry gating strategy for quantifying CXCL9 expression in vivo in tumor cells and endothelial cells.**

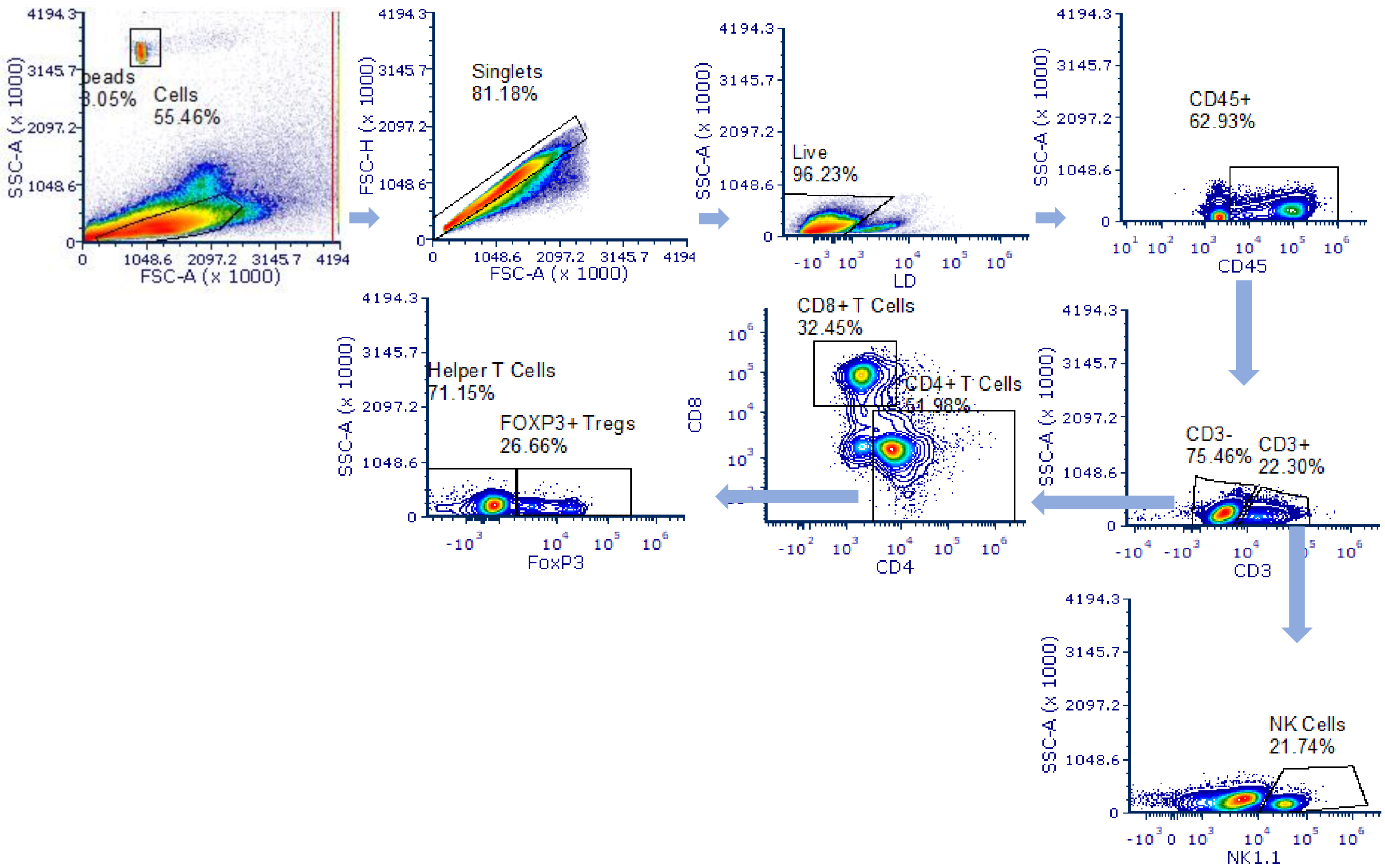


**Figure S8: Flow cytometry gating strategy for profiling immune cell infiltration after FUS-mediated delivery of SH-NPs encapsulating CXCL9-encoding plasmids.**

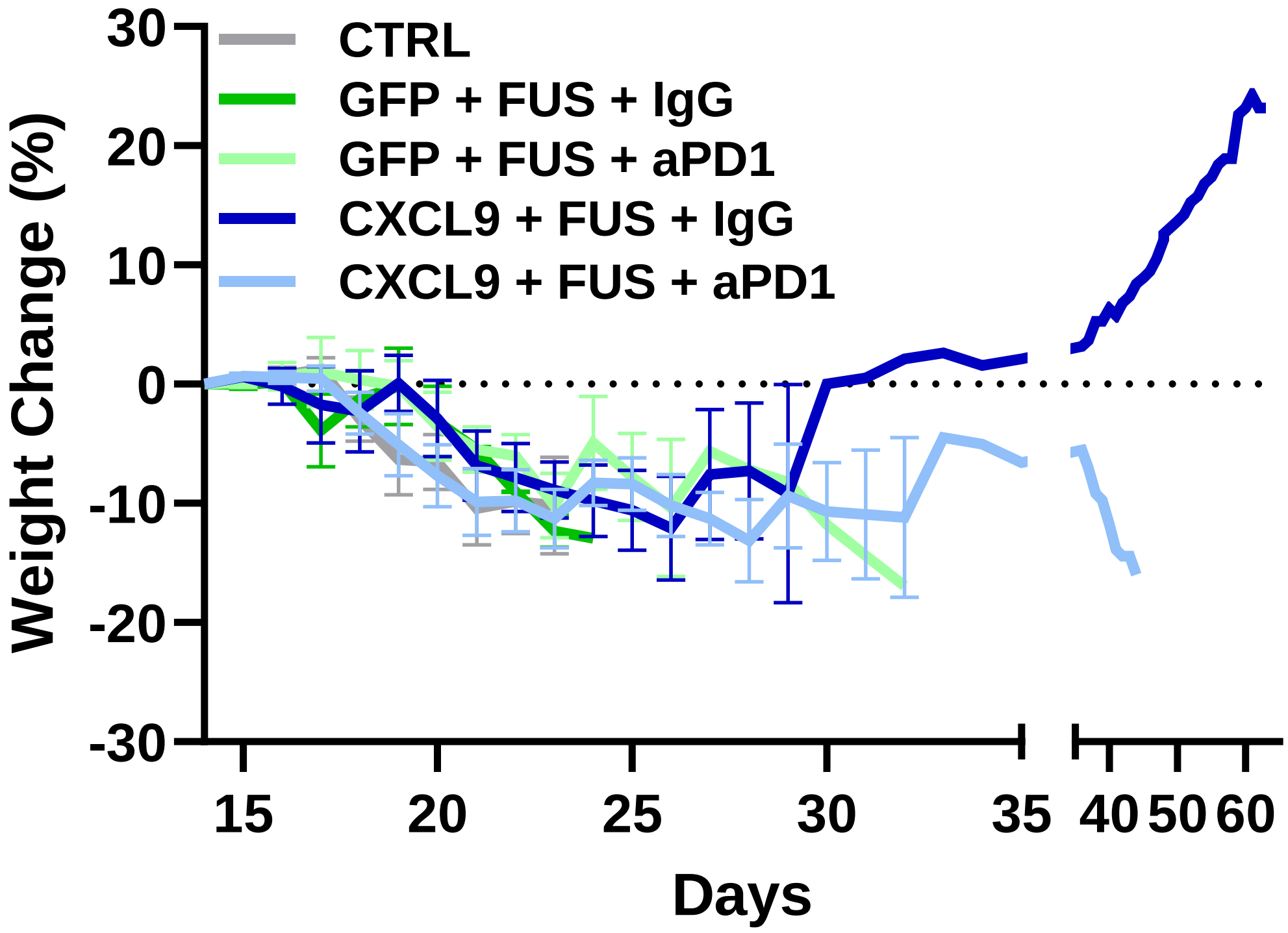


**Figure S9: Mouse weight changes for experiment reported in Figure 4. Means ± S.E.M.**
